# Role of Aryl Hydrocarbon Receptor (AhR) in the Regulation of Immunity and Immunopathology During *Trypanosoma cruzi* Infection

**DOI:** 10.3389/fimmu.2019.00631

**Published:** 2019-03-29

**Authors:** Laura Fernanda Ambrosio, Constanza Insfran, Ximena Volpini, Eva Acosta Rodriguez, Horacio Marcelo Serra, Francisco J. Quintana, Laura Cervi, Claudia Cristina Motrán

**Affiliations:** ^1^Departamento de Bioquímica Clínica, Facultad de Ciencias Químicas, Universidad Nacional de Córdoba, Córdoba, Argentina; ^2^Centro de Investigaciones en Bioquímica Clínica e Inmunología (CIBICI), CONICET, Córdoba, Argentina; ^3^Ann Romney Center for Neurologic Diseases, Brigham and Women's Hospital, Harvard Medical School, Boston, MA, United States; ^4^The Broad Institute of MIT and Harvard, Cambridge, MA, United States

**Keywords:** Chagas disease, TCDD, ITE, regulatory T cells, AhR

## Abstract

Resistance to *Trypanosoma cruzi* infection is dependent on a rapid induction of Th1-type and CD8+ T cell responses that should be promptly balanced to prevent immunopathology. *T. cruzi*-infected B6 mice are able to control parasite replication but show a limited expansion of Foxp3+regulatory T (Treg) cells that results in the accumulation of effector immune cells and the development of acute liver pathology. AhR is a ligand-activated transcription factor that promotes Treg cell development and suppression of pro-inflammatory cytokine production in dendritic cells, altering the course of adaptive immune response and the development of immunopathology. Here, we used different AhR-dependent activation strategies aiming to improve the Treg response, and B6 congenic mice carrying a mutant AhR variant with low affinity for its ligands (AhRd) to evaluate the role of AhR activation by natural ligands during experimental *T. cruzi* infection. The outcome of TCDD or 3-HK plus ITE treatments indicated that strong or weak AhR activation before or during *T. cruzi* infection was effective to regulate inflammation improving the Treg cell response and regularizing the ratio between CD4+ CD25- to Treg cells. However, AhR activation shifted the host-parasite balance to the parasite replication. Weak AhR activation resulted in Treg promotion while strong activation differentially modulated the susceptibility and resistance of cell death in activated T and Treg cells and the increase in TGF-β-producing Treg cells. Of note, *T. cruzi*-infected AhRd mice showed low levels of Treg cells associated with strong Th1-type response, low parasite burden and absence of liver pathology. These mice developed a Treg- and Tr1-independent mechanism of Th1 constriction showing increased levels of systemic IL-10 and IL-10-secreting CD4+ splenocytes. In addition, AhR activation induced by exogenous ligands had negative effects on the development of memory CD8+ T cell subsets while the lack/very weak activation in AhRd mice showed opposite results, suggesting that AhR ligation restricts the differentiation of memory CD8+T cell subsets. We propose a model in which a threshold of AhR activation exists and may explain how activation or inhibition of AhR-derived signals by infection/inflammation-induced ligands, therapeutic interventions or exposure to pollutants can modulate infections/diseases outcomes or vaccination efficacy.

## Introduction

The aryl hydrocarbon receptor (AhR) is a ligand-activated transcription factor that plays important roles in several biological processes, including development, detoxification and the immune response (1). AhR is expressed in immune system cells such as macrophages (Mo), dendritic cells (CDs), NK cells, B lymphocytes and certain subtypes of T cells as Th17 and Treg cells. When inactive, AhR is located in the cytoplasm as part of a protein complex which translocate to the nucleus after ligand activation (1). Genomic AhR signaling pathways involve the interaction of AhR with other transcription factors to directly regulate the transcription of target genes through AhR-responsive elements (AhREs) (1, 2). In immune cells, AhR can be activated by several physiological ligands that include many derivatives of tryptophan (Trp) such as L-kynurenine (Kyn) and 3-hydroxy-kynurenine (3-HK) generated by indoleamine 2,3 dioxygenase (IDO) activity (3, 4) and other endogenous ligands as the Trp photoproducts 6-formylindolo[3,2-b]carbazole (FICZ) and 2-(1*H*-Indol-3-ylcarbonyl)-4-thiazolecarboxylic acid methyl ester (ITE) (5). Also exogenous xenobiotics such as 2,3,7,8-tetrachlorodibenzo-p-dioxin (TCDD) triggers AhR-mediated signaling (5). AhR activation can regulate innate and adaptive immune responses via regulation of multiple AhREs present in the promoter regions of several genes, such as those implicated in the regulation of NF-kappa-B (6) and the development of regulatory T cells (Treg) (Foxp3, TGF-β and IL-10) and Th1 (IL-12), and Th17 (IL-21 and IL-23) cells (1, 2, 7). In particular, AhR has been shown to regulate the inflammatory response at different levels, for example imprinting tolerogenic properties to DCs and promoting the development of regulatory T cells (Tr1, and CD4+CD25+Foxp3+ Treg cells) (7–9). In that way, AhR plays an important role in the regulation of autoimmune inflammatory diseases as rheumatoid arthritis (10), psoriasis (11) and experimental autoimmune encephalomyelitis (EAE) (12–14).

Chagas' disease, caused by the protozoan parasite *Trypanosoma cruzi*, represents a major public health problem in the Americas from Mexico to southern Argentina. Each year there are approximately 12,000 deaths which are attributable to Chagas disease, typically due to severe chronic Chagas disease cardiomyopathy (CCC), which affects approximately 30% of infected individuals and occurs decades after acute infection (15). The remaining patients develop digestive disorders (5–10%) or persist asymptomatic (Asy) and free from cardiac or digestive disorders (60–70%). Although the mechanisms underlying the differential progression to CCC are still not fully understood, it is clear that CCC patients display a more intense inflammation than Asy patients, who appear to have a more regulated immune response. The former show increased circulating levels of pro inflammatory cytokines (16), together with increased numbers of IFN-γ producing T cells and reduced numbers of IL-10-producing CD4+ T cells (17–19) and Treg cells (20–22) in peripheral blood compared with patients having the Asy form of Chagas disease. In addition, a Th1-rich inflammatory infiltrate predominantly secreting IFN-γ and TNF is found in the heart tissue of CCC patients (17, 23–27). Taken together, these results suggest that, in addition to parasite-mediated damage, the unbalanced T cell response plays a role in CCC development.

Host resistance during experimental *T. cruzi* infection is dependent on a rapid induction of a Th1 inflammatory response and CD8+ T cell mediated immunity. Th1 mediators are essential for pathogen control during the acute phase of the infection while CD8+ T cells are important throughout all the stages of the infection, although not sufficient for complete parasite elimination (28). In addition, diverse regulatory mechanisms should promptly balance the inflammatory response to prevent the immunopathology. Studies in which C57BL/6 (B6) mice are infected with the Tulahuén strain of *T. cruzi* revealed an acute disease accompanied by splenomegaly and liver damage (29). Likewise to that observed in CCC patients, B6 mice have great difficulty in controlling the inflammatory response leading to the premature death of these animals by liver failure. In this experimental setting, the increased morbidity was associated to high levels of TNF and low levels of IL-10 (29). Interestingly, it was demonstrated that B6 mice do not expand the population of Treg cells in parallel with the large expansion undergone by the T cell compartment, resulting in an increased ratio of T effector/Treg cells (30, 31). These results suggest that, as observed during human Chagas disease, the fatal outcome in B6 mice may be linked to an unbalanced Th1 response by poor Treg cell induction. In this way, the severity of *T. cruzi*-induced immunopathology may be ameliorated by regulating the balance of CD4+ effector and Treg cells, and procedures that change this balance could represent a promising approach for therapy.

We have previously demonstrated that IDO activity is up-regulated after *T. cruzi* infection in mice, being the Trp catabolite 3-HK toxic for *T. cruzi* amastigotes and trypomastigotes (Tps) (32, 33). We assayed the treatment of *T. cruzi* infected BALB/c mice with 3-HK and observed that, in addition to control the parasite load, the treatment was able to modulate the immune response at the acute phase of the infection impairing the Th1- and Th2-type specific immune response, inducing TGF-β-secreting cells, promoting the emergence of Treg cells and markedly reducing the incidence and the severity of the inflammatory pathology (32, 34). Because there is a well-established connection between inflammation, AhR expression and IDO induction, and considering that Trp-derived IDO-induced catabolites Kyn and 3-HK AhR ligands may signal AhR to promote Treg cell induction (3, 4, 35–37), here, we evaluated the role of physiological AhR signaling and the effect of the treatment with different AhR agonists on the regulation of the immune response and the outcome of *T. cruzi* infection in B6 mice.

## Materials and Methods

### Mice and Parasites

All animal experiments were approved by and conducted in accordance with guidelines of the Animal Care and Use Committee of the Facultad de Ciencias Químicas, Universidad Nacional de Córdoba (Approval Number HCD 743/18). C57BL/6 (B6) was obtained from School of Veterinary, La Plata National University (La Plata, Argentina) and B6.D2N-Ahrd/J (AhRd), kindly provided by Dr. Francisco Quintana (Ann Romney Center, Boston, USA). All animals were housed in the Animal Facility of the Facultad de Ciencias Químicas, Universidad Nacional de Córdoba (OLAW Assurance number A5802-01). The Tulahuen strain of *T. cruzi* was used, which was maintained by weekly intraperitoneal (ip) inoculations in mice.

### Treatments and Parasite Load

Groups of B6 mice (6–8 weeks old) maintained under standard conditions were ip injected with 1 ug of TCDD (AccuStandard, New Haven, CT, USA) or vehicle (DMSO, Sigma-Aldrich) 24 h before to be infected with 50,000 bloodstream trypomastigotes (Tps) of *T. cruzi*. To evaluate the effect of weak AhR activation on acute and chronic phase of the infection, B6 mice were infected with 50,000 *T. cruzi* Tps, and 5 days post-infection (pi), were ip injected with 3-HK (1 mg/kg/day, Sigma Aldrich) for 5 consecutive days (5–10 post-infection) (32, 34) and with ITE (200 ug Tocris Bioscience, R&D Systems) on days 7, 9, and 11 pi. 3-HK and ITE were resuspended in 0.1 M PBS and DMSO, respectively, with these vehicles also being employed as control. AhRd mice and its B6 counterpart were ip infected 50,000 Tps of Tulahuen strain. The levels of parasitemia were monitored in blood collected at different times pi as previously described (32). For determination of tissue parasitism, genomic DNA was purified from liver, heart and skeletal muscle using TRIzol reagent (Life Technologies) and following manufacturer's instructions. Satellite DNA from *T. cruzi* (GenBank AY520036) was quantified by real time PCR using specific Custom Taqman Gene Expression Assay (Applied Biosystem) using the primer and probe sequences described by Piron et al (38). A sample containing 200 ng of genomic DNA was amplified and considered positive for *T. cruzi* when the threshold cycle (CT) for the *T. cruzi* target when C_T_ <45. Abundance of satellite DNA from T. cruzi was normalized to GAPDH abundance (Taqman Rodent GAPDH Control Reagent, Applied Biosystem) and expressed as arbitrary units.

### Transaminase Activity

Plasma aspartate aminotransferase (AST) and alanine aminotransferase (ALT) activities were measured using commercial kits (Wiener Lab) following manufacturer instruction.

### Cell Preparations and Culture

Spleen was obtained and homogenized through a tissue strainer. Erythrocytes in cell suspensions were lysed for 5 min in Tris-ammonium chloride buffer (Sigma Aldrich), washed and resuspended in complete medium, containing RPMI 1640 (Gibco,) supplemented with 2 mM GlutaMAX (Gibco, ThermoFisher), 10 ug/ul gentamicin (Richet S.A, CABA, Argentina) and 10% FBS (NATOCOR, Cordoba, Argentina). Viable cell numbers were determined by trypan blue exclusion using a Neubauer counting chamber. For cytokine determinations, spleen mononuclear cells (SMC) were cultured with or without *T. cruzi* lysate (1 mg/ml) at 2 × 10^6^ cells/ml in complete RPMI medium in 24-well plates (Costar) for 72 h. Supernatants were collected and the secreted cytokines measured.

### Cytokine Quantification

Cytokines were measured in sera and cell culture supernatants by capture ELISA using antibodies and protocols suggested by the manufacturer (eBiociences, ThermoFisher). Cytokine concentration in serum samples was expressed as pg/ml, while the cytokine levels in culture supernatants were represented as Index obtained by dividing the cytokine concentration in supernatant of *T. cruzi*-stimulated cultures/cytokines concentration in supernatants of non-stimulated cultures (medium).

### Flow Cytometry

Cell suspensions were washed in ice-cold FACS buffer (PBS-2% FBS) and incubated with fluorochrome labeled-Abs for 20 min at 4°C. Different combinations of the following Abs were used: anti-CD25-PeCy7 (eBiociences, ThermoFisher), anti-CD25-APC (eBiociences, ThermoFisher), anti-CD4-PerCp5.5 (eBiociences, ThermoFisher), anti-CD44-PECy7 (eBiociences, ThermoFisher), anti-CD62L-APCCy7 (BD Bioscience), anti CD8-FITC (BD Bioscience), anti-FR4-FITC (eBiociences, ThermoFisher), anti-LAP-PeCy7 (eBiociences, ThermoFisher) and an H-2K(b) *T. cruzi* trans-sialidase amino acids 569-576 (ANYKFTLV) (TSKB20) APC-Labeled Tetramer (NIH Tetramer Core Facility), which was used for staining 15 min before the addition of the rest of the surface markers. Intracellular cytokines were detected after stimulating cells during 4 h with 50 nM PMA (Sigma Aldrich) and 0.5 μg/ml ionomycin (Invitrogen, ThermoFisher), or 10 μg/ml *T. cruzi* lysate in the presence of GolgiStop (BD Biosciences). Cells were surface-stained, fixed, and permeabilized with BD Cytofix/Cytoperm and Perm/Wash (BD Biosciences) according manufacturer's instruction and intracellular stained with anti-IL-10-APC (eBiociences, ThermoFisher), anti-IL-10-PE (eBiociences, ThermoFisher), anti-IL-17-PE (eBiociences, ThermoFisher), anti-IFN-γ-APC (eBiociences, ThermoFisher), and anti-Foxp3-PE (eBiociences, ThermoFisher). For Annexin V and 7-AAD staining, the Apoptosis detection kit I (BD Pharmingen) was used. Then, were washed and acquired in FACSCanto II (BD Biosciences). For LAP staining the cells were activated with PMA and ionomycin during 4 h in the absence of GolgiStop.

### Statistical Analysis

Data distribution was analyzed with Shapiro-Wilk test. Normally distributed data were presented as means ± SD. Differences between the mean values were assessed using Student's *t*-test. Differences on survival were analyzed applying Gehan-Breslow-Wilcoxon test. Results were considered significantly different when *p* < 0.05. Statistic was performed using GraphPad Prism7 software.

## Results

### Activation of AhR With TCDD Impairs Host Resistance to *T. cruzi* Experimental Infection

Evidence from studies to control autoimmunity and graft-vs.-host disease, even virus induced inflammatory lesions indicate that the regulation of the immune response to avoid immunopathology could be achieved by the administration of the stable agonist of AhR, TCDD (7, 39–41). TCDD is a non-degradable high affinity ligand for AhR and most studies using this ligand have reported inhibitory effects on inflammatory reactions (40, 41).

**Figure 1:**
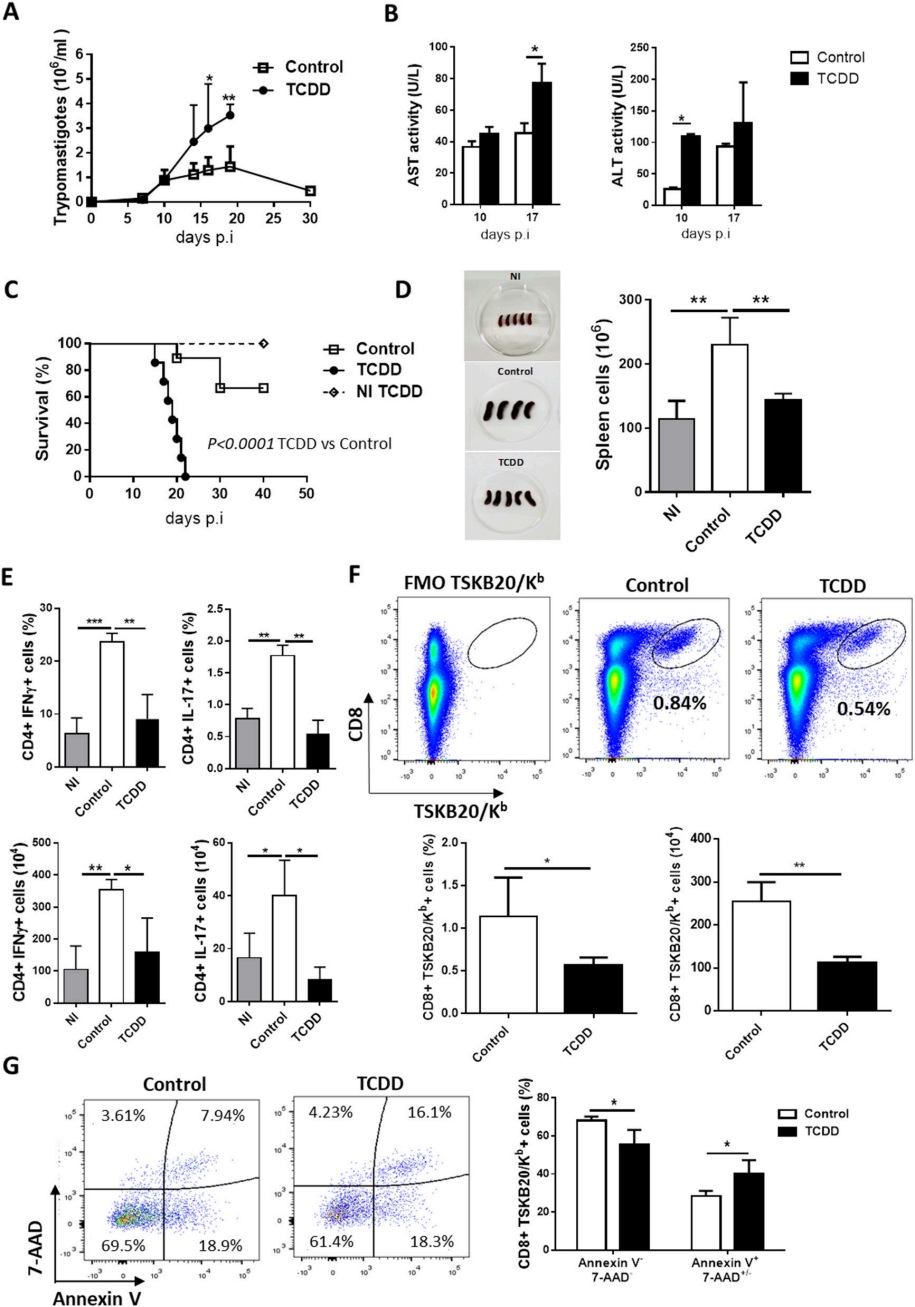
TCDD treatment suppress T cell expansion, cytokine production, and impairs resistance to *T. cruzi* infection. C57BL/6J mice were ip injected with TCDD or DMSO (Control) 24 h before being infected with 50,000 Tps of *T. cruz*i. **(A)** Parasitemia (Tps/ml blood). Data are shown as mean ± SD of 7-9 mice per group. **(B)** ALT and AST activity determined in plasma at day 10 and 17 pi. Data are shown as mean ± SD, *n* = 3 mice per group. **(C)** Survival rate of non-infected TCDD treated (NI TCDD), infected TCDD-treated and infected vehicle-treated (control) mice. *P*-values were calculated with the Gehan-Breslow-Wilcoxon test. **(D)** Images of spleens from NI and infected TCDD-treated and control mice in one representative experiment. The bars represent the number of total spleen cells from NI and infected TCDD-treated and control mice ± SD. **(E)** Splenocytes taken from NI (*n* = 4) and *T. cruzi*-infected mice treated (TCDD, *n* = 5) or not (Control, *n* = 4) with TCDD at day 10 pi were *in vitro* stimulated with PMA/Ionomycin in the presence of Golgi stop. The bars represent the percentage and absolute number of CD4+ splenocytes producing IFN-γ or IL-17 cytokines. **(F)** Representative dot plots (top) and bars showing the percentage and absolute number (bottom) of splenocytes CD8+ TSKB20/Kb+ from control and TCDD-treated mice at day 10 pi. **(G)** Representative dot plots (left) and bars (right) showing the percentage of Annexin V- 7-AAD- and Annexin V+ 7AAD+/- splenic cells within the CD8+ TSKB20/Kb+ population at day 10 pi. Bars represent the mean values ± SD. Data in A-C and in D–G are representative of three and two independent experiments, respectively. *P*-values were calculated with two-tailed Student's *t*-test. *p < 0.05, **p < 0.01, ***p < 0.001.

To evaluate the role of AhR engagement by TCDD on the outcome of *T. cruzi* infection, a single intraperitoneal (ip) administration of TCDD was given to mice 1 day prior to infection and the effects on parasite load, host survival and protective immune response were compared with vehicle-treated controls. TCDD treatment impaired resistance to infection as treated mice showed increased parasitemia and serum levels of ALT and AST that resulted in decreased survival compared to control mice (Figures 1 A–C). In addition, the treatment with TCDD did not affect survival or adversely affect hepatic enzymes levels in uninfected mice (Figure 1C and data not shown), suggesting that the dose of TCDD used did not present toxic effects per se.

To measure the consequences of TCDD treatment on the specific immune response, mice were sacrificed during the acute phase of infection (day 10 post-infection). At the time of sacrifice TCDD-treated mice showed spleens markedly decreased in size and cell numbers compared with non-treated infected mice, which showed the characteristic infection-induced splenomegaly (Figure 1D).

To determine the effect of TCDD treatment on Th cell polarization, SMC from treated and control mice were stimulated *in vitro* for 4 h with PMA and ionomycin to enumerate cells that produced either IFN-γ or IL-17. In addition, the frequencies of CD8+ T cells specific for the immunodominant epitope TSKB20 (ANYKFTLV) (42) and Foxp3+ CD25+ CD4+ Treg cells were also evaluated. Compared with non-infected controls, *T. cruzi* infected mice showed significantly increased percentages and absolute numbers of CD4+ cells producing IFN-γ and IL-17 while the TCDD treatment resulted in marked decrease in the percentage and absolute number of these effector cell subsets (Figure 1E). In addition, TCDD-treated mice showed a significant reduction of specific CD8+ TSKB20/Kb+ cells (Figure 1F).

To investigate whether TCDD treatment differentially affected cell survival of infection-activated *T. cruzi*-specific T cells, we compared the level of cell death in the CD8+ TSKB20/Kb+ population from TCDD-treated and control mice by assaying Annexin V binding and 7-AAD permeability. *T. cruzi*-specific CD8+ T cells from TCDD-treated mice showed lower frequency of live cells (Annexin V-, 7-AAD-) and higher frequency of dead cells (Annexin V+, 7-AAD+/-) than those from control mice (Figure 1G), suggesting a direct toxic effect of TCDD on parasite-activated cells.

**Figure 2:**
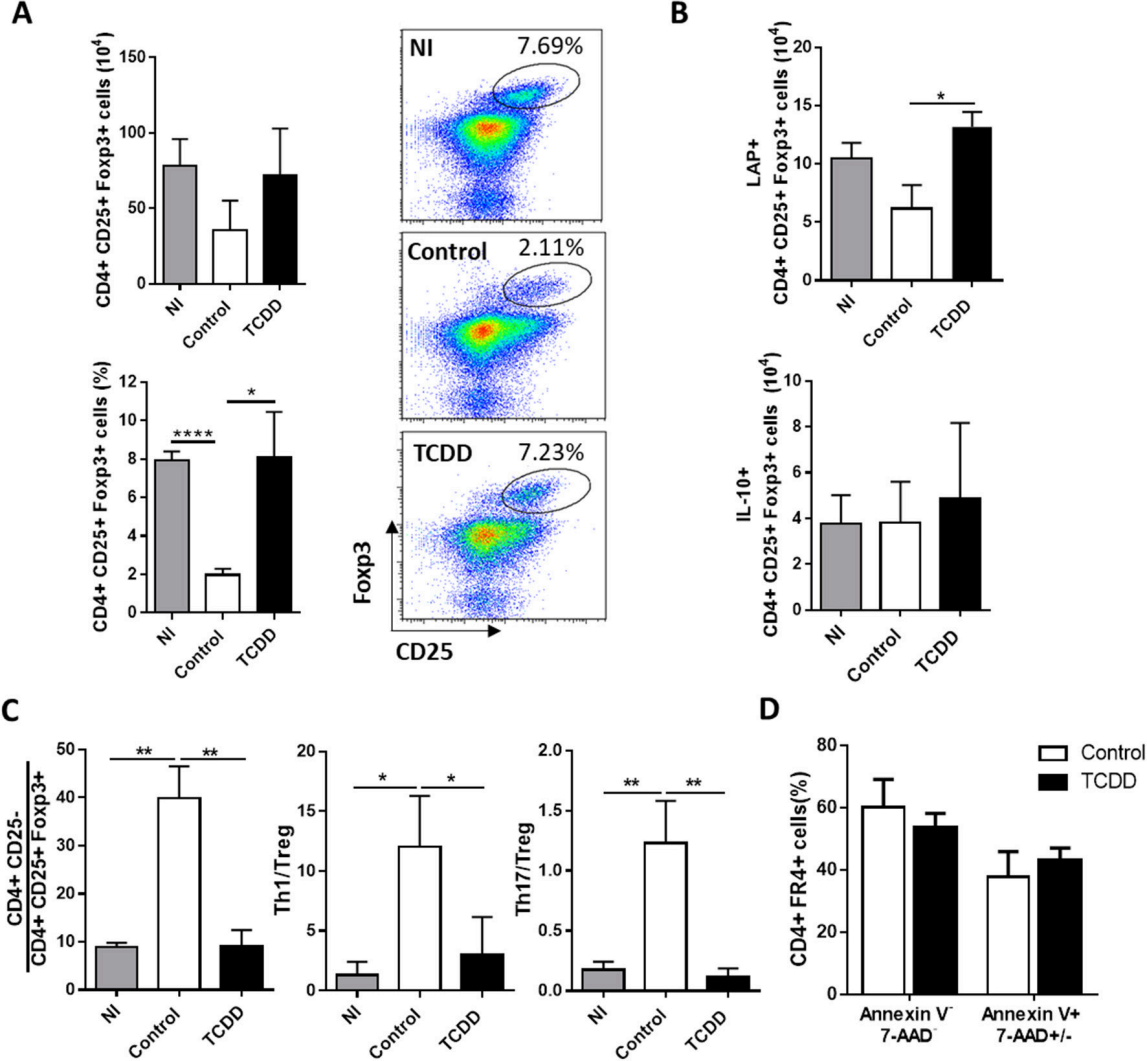
TCDD treatment shifted the balance between inflammatory and CD4+ CD25+ Foxp3+ Treg cells**. (A)** Representative dot plots (right) and bars (left) showing the percentage and absolute number of splenocytes from NI, control and TCDD-treated mice expressing CD25+ Foxp3+ within CD4+ population at day 10 pi. Bars are shown as mean ± SD, *n* = 3 mice per group. **(B)** Absolute number of CD4+ CD25+ Foxp3+ IL-10+ and CD4+ CD25+ Foxp3+ LAP+ splenocytes after PMA/Ionomycin stimulation at day 10 pi. Bars are shown as mean ± SD, *n* = 3 mice per group **(C)** Ratio of splenic effector T cells (CD4+CD25-), Th1 cells (CD4+Foxp3-IFNγ-) and Th17 cells (CD4+ IL-17+) to Treg cells (CD4+CD25+Foxp3+). Bars represent mean values ± SD with *n* = 3 mice per group. **(D)** Percentage of Annexin V- 7-AAD- and Annexin V+ 7AAD+/- spleen cells within the CD4+ FR4+ population. Data in A-C and in D are representative of three and two independent experiments, respectively. *P-*values were calculated with two-tailed Student's *t*-test. *p < 0.05, **p < 0.01, ****p < 0.0001.

When the effect of TCDD treatment on CD4+ CD25+ Foxp3+ Treg population was evaluated, it was observed that although the numbers of splenic Treg cells were not significantly different between both experimental groups, the percentage of Treg cells and the number of Treg cells producing the immunoregulatory cytokine TGF-β were significantly higher in TCDD-treated compared to control mice, while no differences were observed for IL-10-secreting Treg cells (Figures 2A,B). In addition, TCDD treatment decreased the ratio between total numbers of CD4+CD25- (conventional CD4+ T cells), Th1 cells (IFN-γ-producers) and Th17 cells (IL-17-producers) and total numbers of Treg cells (Figure 2C). Of note, TCDD treatment normalized the ratio of total and effector cytokine-producing conventional CD4+ T cells and Treg cells to values similar to those observed in uninfected mice (Figure 2C). These findings suggested that Treg cells are much less sensitive to TCDD-induced death than conventional T cells. To evaluate this hypothesis, we analyzed comparatively the level of cell death in the CD4+ FR4^hi^ cell population [phenotype compatible with Treg cells (43)] from treated and control mice. Figure 2D shows that the level of cell death in the Treg cell population from TCDD-treated and control mice was similar.

Given the results described above, TCDD might be effective to induce regulation of the inflammatory response in *T. cruzi* infected B6 mice but would not be recommended for usage during the induction of the immune response triggered by the initial parasite replication. With this in mind, we performed additional experiments in which TCDD treatment was given on day 7 pi, a time point in which the innate and effector T cell response is ongoing and the inflammation-induced damage may begin to become apparent. Although TCDD administration on day 7 pi prolonged the survival of TCDD-treated mice, it still increased mice mortality in comparison to controls (not shown).

### Combined AhR Agonists Promote Treg Cell Differentiation and Have Detrimental Effects on Parasite-Specific Immunity

**Figure 3:**
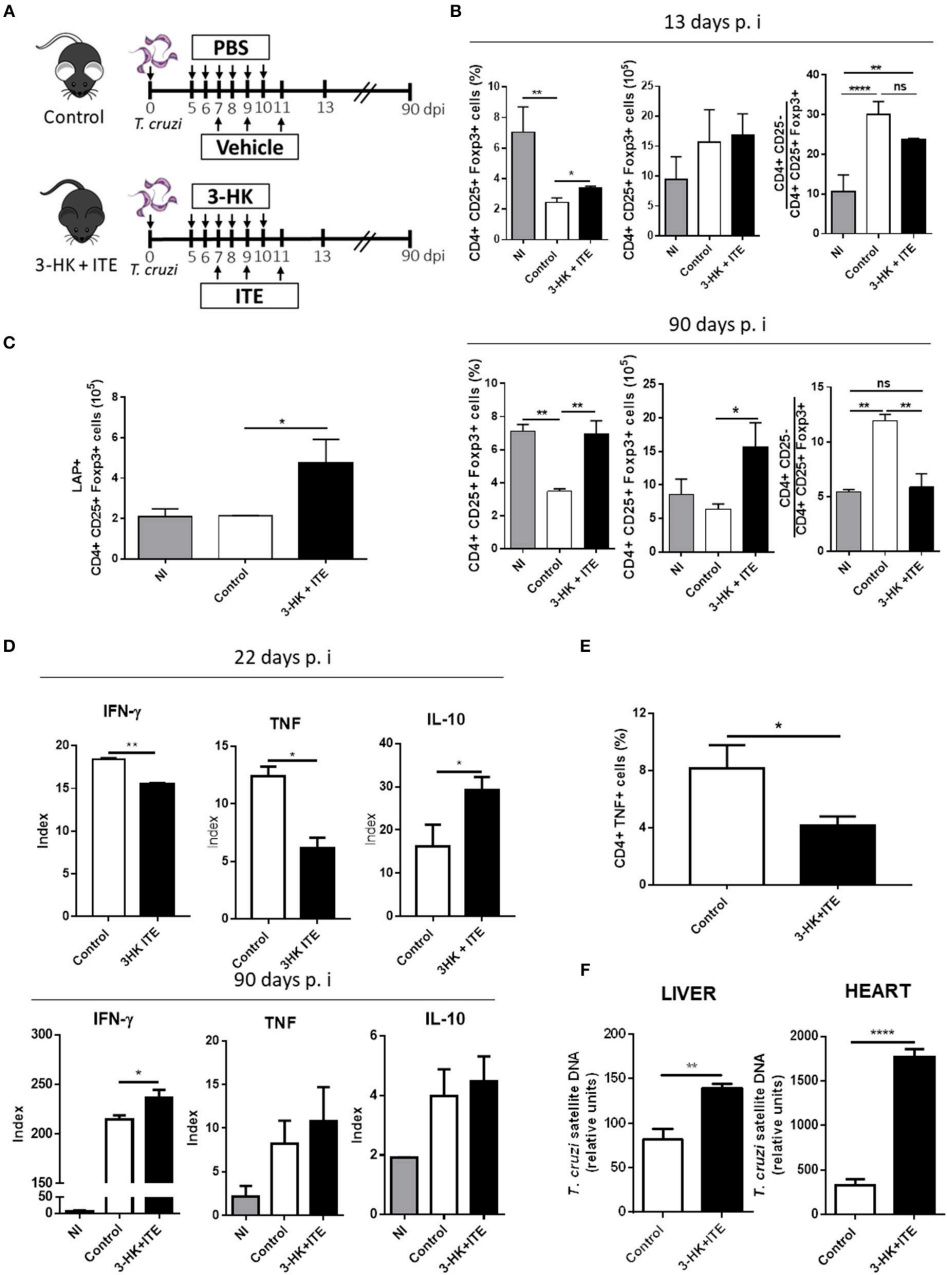
3-HK plus ITE treatment promotes the differentiation of Treg cells with detrimental effects on protective immune response. **(A)** *T. cruzi-*infected B6 mice were treated with 3-HK plus ITE with vehicle-treated mice used as control (PBS+DMSO). **(B)** Percentage, absolute number of splenic CD25+ Foxp3+ cells within CD4+ population and ratio of effector T cells (CD4+CD25-) to Treg cells (CD4+CD25+Foxp3+) at days 13 and 90 pi. Bars are shown as mean ± SD with *n* = 3 mice per group. **(C)** Absolute number of splenic CD4+ CD25+ Foxp3+ LAP+ cells at 90 days pi after PMA/Ionomycin stimulation. Bars are shown as mean ± SD with *n* = 3 mice per group. **(D)** Cytokine levels assayed in supernatants of splenocytes taken at 22- and 90-days pi and cultured with medium alone or total *T. cruzi* lysate for 72 h. The results are represented as an Index which is the ratio between cytokine concentration in supernatant of *T. cruzi*-stimulated cultures and cytokine concentration in the corresponding not stimulated culture. **(E)** Percentage of splenic cells CD4+ TNF+ cells at 22 days pi after 4 h of PMA/Ionomycin stimulation. **(F)** Relative amount of *T. cruzi* satellite DNA in heart and liver at day 90 pi. GAPDH was used as endogenous control for normalization. One experiment representative of two independent is shown. *P-*values were calculated with two-tailed Student's *t*-test. *p < 0.05, **p < 0.01, ****p < 0.0001.

As TCDD, the endogenous AhR ligand ITE is able to induce Treg cells (12, 44, 45), but is nontoxic and could, therefore, be useful to modulate the inflammatory response without a cytotoxic effect on parasite-specific T cells. Furthermore, it has been demonstrated that 3-HK is a weak AhR agonist (3) and also it is active against the amastigote and trypomastigote forms of *T. cruzi* (32, 34). Considering the background mentioned above, we developed a treatment scheme to simultaneously activate AhR and inhibit *T. cruzi* replication that consisted in the daily administration of 3-HK from days 5 to 10 pi together with ITE injection at days 7, 9, and 11 pi, as depicted in Figure 3A. The cellular immune response and the levels of parasite load in the target tissues were then investigated. Acutely infected mice treated with 3-HK plus ITE did not show differences in parasitemia and survival compared with control mice (Figure S1 in Supplementary Material). However, *T. cruzi*-infected B6 mice treated with ITE plus 3-HK had a significant increase in the % (day 13 and 90 pi) and number (day 90 pi) of splenic Treg cells (Figure 3B), and in the number of Treg cells producing TGF-β (day 90 pi) (Figure 3C) when compared with controls. Moreover, 3-HK plus ITE treatment reversed the characteristic unbalanced ratio between conventional CD4+ T cells to Treg cells to values like those observed in uninfected mice (Figure 3B).

To evaluate the cellular immune response developed in 3-HK plus ITE-treated mice, the Ag-specific cytokine production was analyzed in SMC taken at different times pi after *ex vivo* restimulation with parasite lysate. *T. cruzi*-stimulated SMC taken from mice treated with 3-HK plus ITE at the acute phase of the infection (day 22 pi) secreted significantly lower amounts of TNF and IFN-γ and higher amounts of IL-10 than those from untreated mice (Figure 3D). In agreement, treated mice showed a significant lower percentage of TNF-producing spleen CD4+ T cells than control mice (Figure 3E). In addition, only a small increase in the production of IFN-γ by *T. cruzi*-stimulated SMC was observed in treated vs. control mice during the chronic phase of the infection (90 days pi).

Finally, to determine whether the 3-HK+ITE-induced regulation of the specific response compromised the protective immunity to *T. cruzi*, the parasite load was determined in liver and heart at 90 days pi. Compared with untreated, the mice that had been treated with 3-HK plus ITE showed higher parasite load in both liver and heart (Figure 3F). This increased parasite burden could underlie the higher production of IFN-γ by SMC from chronically infected 3-HK plus ITE-treated mice.

Together, our results suggest that 3HK plus ITE might be a novel therapeutic treatment able to control the inflammatory response. However, considering our results and a recent report (31) the expansion of Treg cells during the acute phase of *T. cruzi* infection with *T. cruzi* may also prevent the emergence of protective anti-parasite immunity and critically influence host resistant.

### AhRd Mice Develop a Proper Inflammatory and Anti-inflammatory Response Able to Restrict Parasite Replication

**Figure 4:**
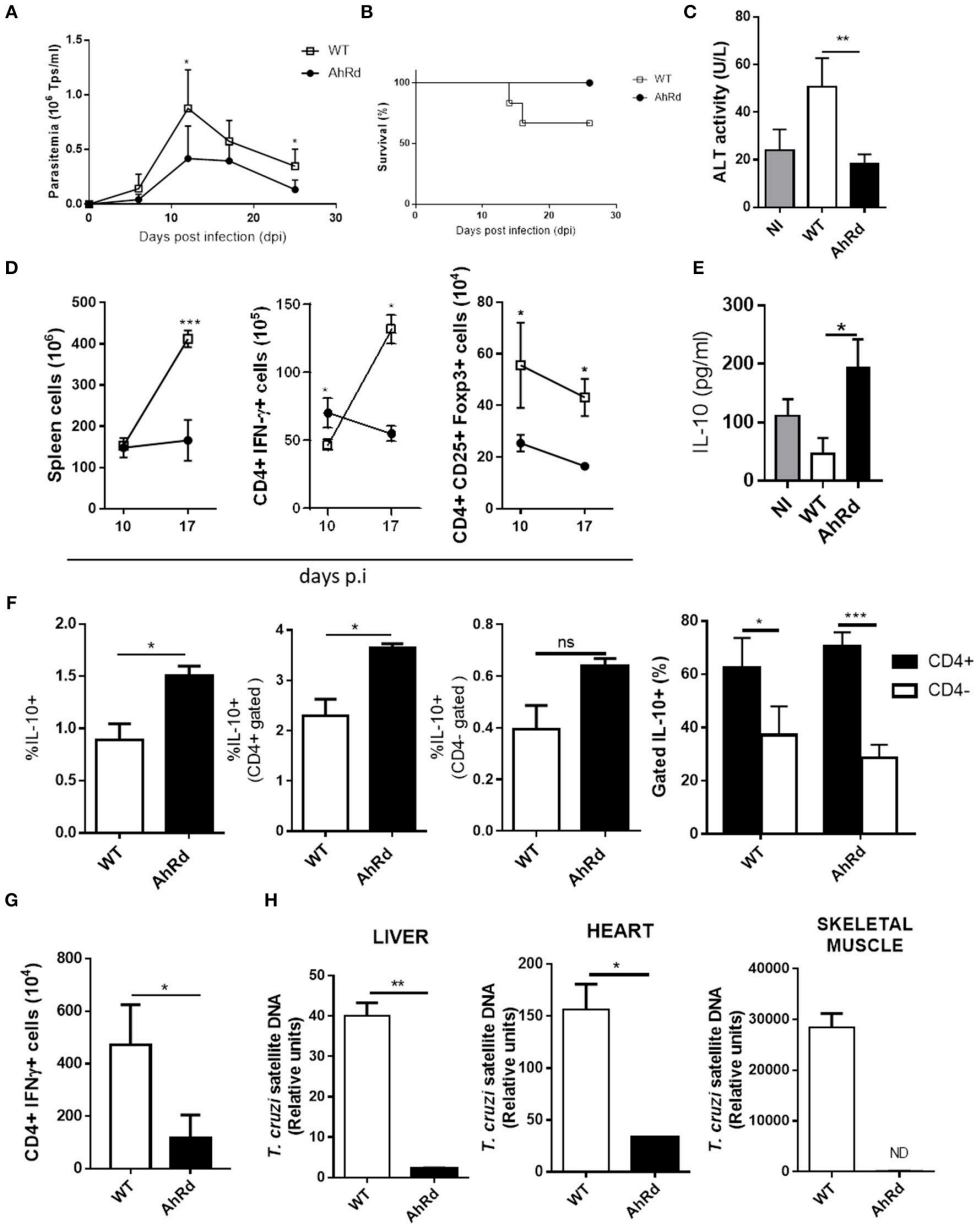
AhRd mice develop a proper inflammatory and anti-inflammatory response able to restrict parasite replication. AhRd and WT mice infected with 50,000 Tps of *T. cruzi* were evaluated for parasitemia, survival and other groups sacrificed at days 10, 17, and 170 pi. **(A)** Parasitemia (Tps/ml blood). Data are shown as mean ± SD, *n* = 5 mice per group **(B)** Survival rate of *T. cruzi* infected WT and AhRd mice, *n* = 5 mice per group. **(C)** ALT activity determined in sera at day 17 pi. **(D)** Absolute number of CD4+ IFNγ+ and CD4+ CD25+ Foxp3+ cells in spleen from WT- and AhRd-infected mice at day 10 and 17 pi. **(E)** Levels of IL-10 in sera from NI, and infected WT and AhRd mice at day 10 pi. **(F)** Percentage of total, CD4+ and CD4- splenocytes that produce IL-10 after PMA/Ionomycin stimulation at day-17 pi. **(G)** Absolute number of CD4+ IFNγ-producing splenocytes at day 170 pi after *in vitro* stimulation with *T. cruzi* total lysate (10 ug/ml). **(H)** Relative amount of *T. cruzi* satellite DNA in heart, liver and skeletal muscle from WT and AhRd mice at day 170 pi. GAPDH was used as endogenous control for normalization, *n* = 4 mice per group. One experiment representative of three independent experiments is shown. *P* values were calculated with two-tailed Student's *t*-test. *p < 0.05, **p < 0.01, ***p < 0.001.

To investigate further on the role of AhR activation during the *T. cruzi* infection and considering that endogenous AhR ligands are generated during this infectious process, we took advantage of AhRd mice, a congenic B6 mice expressing a mutant AhR protein with reduced affinity for its ligands (46). AhRd and WT mice were infected and parasitemia, tissue parasite load, survival and the splenic T cell populations were studied during the acute and the chronic phase of the infection. Infected AhRd mice presented significantly lower peak of parasitemia (Figure 4A), had prolonged survival compared to WT mice (Figure 4B) and displayed significantly lower levels of the hepatic transaminase ALT in sera (Figure 4C). AhRd mice also showed at day 10 pi an expansion of CD4+ IFN-γ producing cells compared with WT mice and (Figure 4D), somehow contrasting previously reported association of high Th1-type response and liver tissue damage in B6-infected mice (29). In addition, we found a strong increase in the number of splenic as well as IFN-γ-producing cells between days 10 and 17 pi in WT, but not in AhRd, mice (Figure 4D). As expected, and consistent with the low strength of AhRd signaling (3, 7), the number of splenic Treg cells was significantly lower in AhRd compared to WT mice (Figure 4D). Also, B6- and AhRd-infected mice showed similar levels of CD4+ IL-17-producing cells (not shown). Interestingly, whereas the levels of pro-inflammatory cytokines in sera were similar in both mice groups (data not shown), the levels of IL-10 in sera and the % of IL-10-producing cells were significantly higher in AhRd vs. WT mice during the acute phase of the infection (Figures 4E,F), being a CD4+ cell population the main producer of this cytokine at day 10 pi (Figure 4F). In addition, AhRd mice exhibited lower number of parasite-specific IFN-γ-producing splenocytes during the chronic phase of the infection, in concordance with lower parasite load in liver, heart and skeletal muscle compared with WT mice (Figures 4G,H). Taken together, these results indicate that AhR signaling is critically involved in the development of robust parasite-specific Th1 responses and immunoregulatory mechanisms during *T. cruzi* infection.

### AhR Signaling Restricts the Differentiation of Memory CD8+ T Cell Subsets During *T. cruzi* Infection

**Figure 5:**
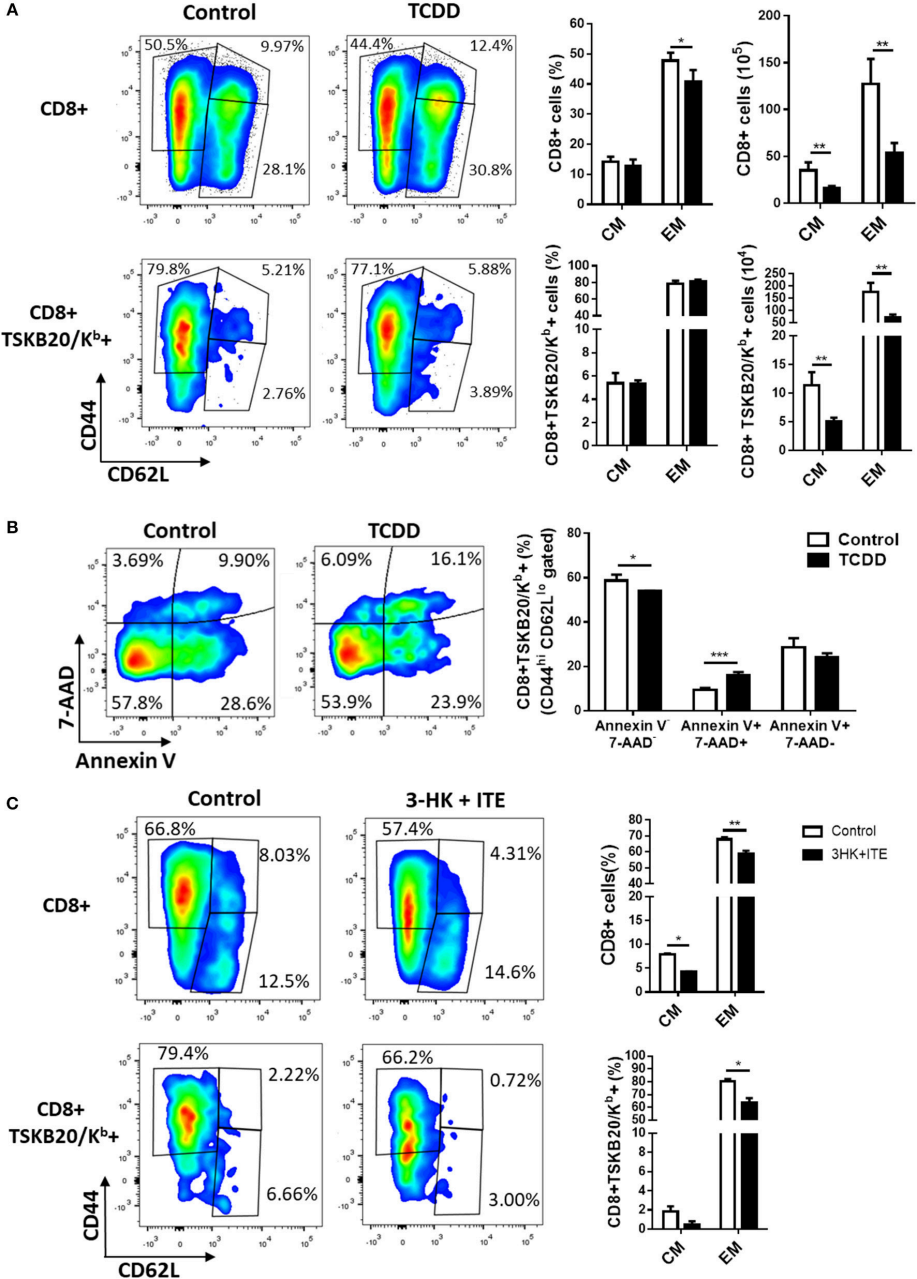
AhR ligation regulates the development of memory CD8+ T subsets during *T. cruzi* infection. Splenocytes from TCDD- and 3HK + ITE-treated mice and its respective controls were isolated at day 10- and 22-days pi to evaluate total CD8 and TSKB20/K^b^-specific memory subpopulations by flow cytometry. **(A)** Representative plots, percentage, and absolute number of splenocytes from control and TCDD-treated mice at day 10 pi showing effector/memory (EM, CD44+CD62L−) and central memory (CM, CD44+CD62L+) phenotype on CD8+ and CD8+ TSKB20/K^b^+ cell populations gated as described in Figure S2A in Supplementary Material. Bars represent the mean values ± SD. Control, *n* = 5 and TCDD, *n* = 4. **(B)** Representative plots and bars showing the percentage of splenocytes expressing Annexin V- 7-AAD-, Annexin V+ 7AAD- and Annexin V+ 7AAD+ cells within the CD8+ TSKB20/K^b^+ EM cell population of Control and TCDD treated mice at day 10 pi. Bars represent the mean values ± SD. Control, *n* = 5 and TCDD, *n* = 4. **(C)** Representative plots and bars showing the percentage of splenocytes from control and 3-HK plus ITE-treated mice with EM and CM phenotype within CD8+ and CD8+ TSKB20/K^b^+ cell populations (gated as indicated in Figure S2B of Supplementary Material) at day 22 pi. Bars represent the mean values ± SD. Control, *n* = 3 and 3-HK+ITE, *n* = 3. One experiment representative of three independent experiments is shown. *P-*values were calculated with two-tailed Student's *t*-test. *p < 0.05, **p < 0.01, ***p < 0.001.

The acquisition of memory T cells is defined by the generation and persistence of T cells that can provide long-lasting protection against pathogens. Signals given by DC as TCR engagement, costimulation and cytokines co-participate in the induction of memory T cells. Thus, changes in any of the factors controlling the activation of T cells during the antigen presentation can regulate T effector and memory cell differentiation. Different observations suggest that AhR, directly or through its effects on antigen presenting cells modulates critical events in the activation of naive T cells that could modify the development of effector and memory T cells (47, 48). To evaluate the role of AhR activation on T cell differentiation of different memory subsets, we evaluated the effect of different strength of AhR ligation on the frequencies of CD8+ total and specific (TSKB20/Kb+) cell subsets defined by CD62L and CD44 expression. Figure 5A shows that strong AhR ligation with TCDD significantly decreased the frequency of CD8+ CD44^hi^ CD62L^lo^ cells (effector/effector memory cells, EM) and the number of both EM and CD8+ CD44^hi^ CD62L^hi^ cells (central memory cells, CM) in spleen at day 10 pi. Moreover, the number of splenic *T. cruzi*-specific (TSKB20/Kb+) EM and CM subpopulations showed a significant decrease in treated compared with control mice. These results suggest a negative effect of a strong AhR activation on the development of effector and memory CD8+ T cells precursors. However, the fact that antigen-experienced cells suffer the direct effect of TCDD toxicity (Figure 1G), does not allow to conclude clearly on the consequence of AhR ligation on the development of memory precursor cells. Indeed, CD8+ TSKB20/Kb+ cells with EM phenotype from TCDD-treated mice showed higher percentage of Annexin V+ 7-AAD+ cells than those from control mice (Figure 5B).

To test the hypothesis that the activation of AhR might alters the *in vivo* distribution and frequency of CD8+ total and parasite-specific T cells subsets, we analyzed the effector phase of memory induction in *T. cruzi* infected B6 mice that were treated with 3-HK plus ITE as depicted in Figure 3A. Figure 5C shows that weak AhR ligation with 3HK plus ITE during *T. cruzi* infection decreased the percentage of total and specific CD8+ splenic T cells that acquired EM and CM phenotype.

**Figure 6:**
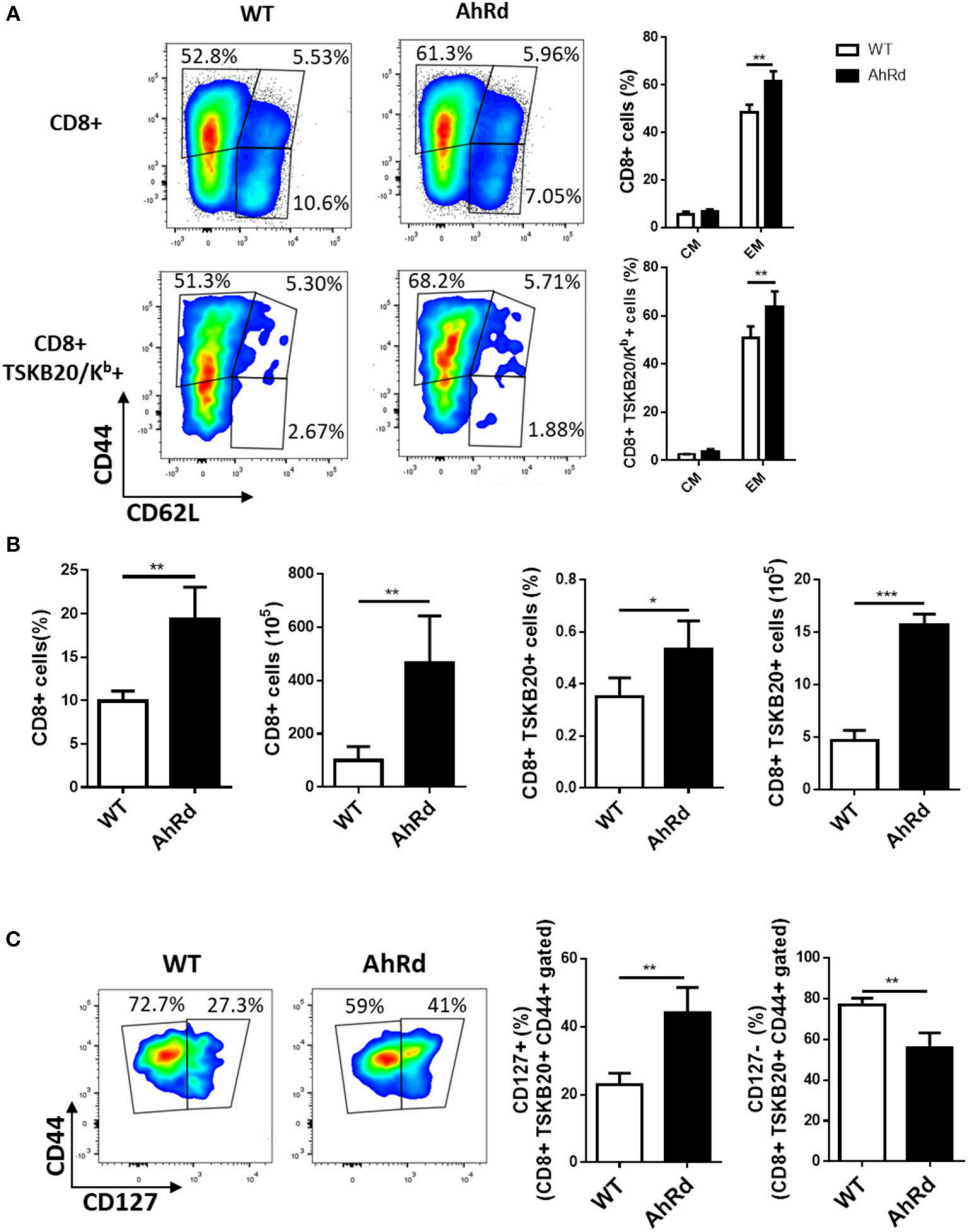
AhR activation restricts the differentiation of CD8+ EM cells. Splenocytes from infected AhRd and WT mice were isolated at day 17 and 170 pi to evaluate total CD8 and TSKB20/K^b^-specific memory subpopulations by flow cytometry. **(A)** Representative plots and bars showing the percentage of splenocytes from WT and AhRd mice bearing EM and CM phenotype on CD8+ and CD8+ TSKB20/K^b^+ T cell populations at day 17 pi. Bars represent the mean values ± SD with WT, *n* = 3 and AhRd, *n* = 4. **(B)** Percentage and absolute number of splenocytes CD8+ and CD8+ TSKB20/K^b^+ from WT and AhRd mice at day 170 pi. Bars represent the mean values ± SD with WT, *n* = 4 and AhRd, *n* = 4. **(C)** Representative plots showing CD44 and CD127 expression on splenocytes from WT and AhRd mice gated on CD8+ TSKB20/K^b^+ cell populations at day 170 pi gated as indicated in Figure S3 of Supplementary Material. Percentage of splenocytes from WT and AhRd mice showing CD127+ and CD127- expression within CD8+ TSKB20/K^b^+ CD44+ cell populations. Bars represent the mean values ± SD with WT, *n* = 4 and AhRd, *n* = 4. One experiment representative of at least three independent experiments is shown. *P-*values were calculated with two-tailed Student's *t*-test. *p < 0.05, **p < 0.01, ***p < 0.001.

To support these findings, we compared the frequency and number of total and *T. cruzi*-specific CD8+ T cells with EM and CM phenotype in AhRd and WT mice after 17 and 170 days pi. Similar frequency and number of CD8+ T cells specific for the immunodominant epitope TSKB20 were observed for AhRd and WT mice at day 20 pi (not shown). Moreover, and in contrast to that observed when AhR was activated by exogenous ligands (Figures 5A,C), the frequency of CD8+ total and *T. cruzi*-specific T cells with EM phenotype was higher in AhRd than WT mice at 17 days pi (Figure 6A) and during the chronic phase of the infection (day 170 pi) (not shown). Interestingly, AhRd mice showed higher percentages and absolute number of CD8+ total and *T. cruzi*-specific T cells than WT mice at the chronic phase of the infection (day 170 pi) (Figure 6B). Analysis of CD44 and CD127 expression, that distinguish central memory cells (CD44^hi^ CD127^hi^) from effector memory cells (CD44^hi^ CD127^lo^), in subpopulations of CD8+TSKB20/Kb+ T cells revealed that the frequency and number of splenic cells with CM phenotype was significantly higher in AhRd than in WT mice during the chronic phase of the infection (Figure 6C and not shown). Taken together, these results suggested that during *T. cruzi* infection physiological AhR ligation restricts the differentiation of CD8+ memory T cells impacting in the magnitude of the long-term parasite-specific immune response and the chronic control of tissue parasitism.

## Discussion

It has been demonstrated that AhR activation can be induced by diverse ligands in the environment, however, Trp metabolites have emerged as key family of physiological agonistic ligands (49). During *T. cruzi* infection, the up-regulation of the enzyme IDO tht catalyzes the Trp degradation along the Kyn pathway has at least two main roles, the control of pathogen growth by producing the catabolite 3-HK and the immune regulation facilitated by the 3-HK-mediated expansion of Treg cells (32, 34). IDO induction depends on AhR expression, and Kyn as well as 3-HK are both AhR ligands that signal for Treg induction (3, 4, 35). Thus, the AhR agonist 3-HK used as a therapy in *T. cruzi*-infected BALB/c mice was able to increase the Treg population and to modify the outcome of the infection (34).

After *T. cruzi* infection, B6 mice develop an important Th1-type response able to control the parasite replication (29). However, and likewise to that observed in CCC patients, *T. cruzi*-infected B6 mice have great difficulty in controlling the inflammatory response, resulting in the premature death by liver failure, being the increased morbidity associated to high levels of TNF and low levels of IL-10 in sera and the incapacity to expand Treg population (29–31). It is known that immune responses can be enhanced or dampened by differential manipulation of Treg cells vs. other naive or activated T cells. In this study, we have assayed different AhR activation-dependent strategies aiming to specifically boost Treg cells in B6 mice infected with *T. cruzi*. The results obtained by TCDD treatment indicated that strong AhR activation during *T. cruzi* infection can normalize the ratio between Th1 and Th17 to Treg cells. Our results showed that the balance of the inflammatory response observed after TCDD treatment was the result of the increased death of activated T cells, the increase in the number of Treg cells producing the immunoregulatory cytokine TGF-β and the resistance of these cells to TCDD-induced death, with no significant changes in the frequency or number of IL-10 producing cells between control and treated mice. It is worth to mention that the direct toxic effect of TCDD on activated T cells has been previously demonstrated (50). In addition, Winans et al. (51) have shown that CD8+ T cells exposed to TCDD exhibit methylation patterns similar to those of exhausted CD8+ T cells suggesting that a strong AhR ligation has an important impact on basal functionality and initial responses to antigenic stimulation, and also explains the decreased memory cell subsets showed in this paper for *T. cruzi*-infected TCDD-treated mice. As far as we know, the resistance to TCDD toxicity by Treg cells has not been described. Taking into account that murine and human Treg cells are more resistant than activated non-Treg CD4+ T cells to apoptosis induced by different pathways such as irradiation, Fas ligation and viral infection (52–55), it should not be surprising that these cells would be also resistant to strong AhR activation. Thus, strong AhR activation with TCDD was effective to regulate inflammation in *T. cruzi* infected B6 mice, but the TCDD-induced immunosuppression contributed to parasite replication, and the treatment resulted in increased parasitemia and death of the treated mice.

The other strategy used to induce Treg cells in B6 mice was to treat the mice with two weak AhR ligands, ITE and 3-HK, to simultaneously activate AhR and concomitantly inhibit parasite replication (32–34). Once more, although this treatment was able to induce Treg cells and improve the unbalanced ratio between CD4+ CD25- to Treg cells during the chronic phase of the infection, the fact that 3-HK is only partially efficient in controlling the parasitemia and unable to eradicate the parasite (32), shifted the host-parasite balance to the parasite replication. Indeed, although 3-HK plus ITE-treated and control mice did not have significant differences in parasitemia and survival during the acute phase of the infection, treated mice showed higher parasite load in both liver and heart during the chronic phase. Considering that TNF and IFN-γ are both critical cytokines to mediate protection while IL-10 mediates susceptibility in *T. cruzi* infection (56), the increase in parasite load was in line with the lower IFN-γ and TNF and higher IL-10 production by splenocytes from 3-HK plus ITE treated mice during the acute phase of the infection.

Thus, the results obtained by using two different experimental strategies to promote Treg development by AhR activation demonstrated that the increase of the number of Treg did not ameliorate the immunopathology issue, since limited Treg response during *T. cruzi* infection in B6 mice is critically required to allow the development of protective parasite specific T cell immunity, as was recently reported by Araujo et al. (31). Decrease host resistance to a variety of infectious agents has been reported when TCDD exposure occurs prior to infection, however, whether the AhR ligation contributes to improve or worsen host resistant depends on the pathogen (48). Thus, in contrast to what is observed for *T. cruzi* infection, AhR activation by TCDD in *Leishmania major* infection reduces the parasite burden (57, 58).

It has also been reported that AhR signaling under Tr1-skewing conditions (IL-27 or TGF-β plus IL-27) promotes the differentiation of Tr1 cells (CD4+ IL-10+) (59). However, neither TCDD nor 3-HK plus ITE treatments were able to significantly increase Tr1 population [CD4+, Foxp3-, IL-10+ Lag-3+ cells (60)] in *T. cruzi-*infected B6 mice (data not shown), in spite of both treatments up-regulated TGF-β production and *T. cruzi* infection induces IL-27 production in B6 mice (30).

By using AhR-/- (KO) mice, Barroso et al. have examined the role of AhR in the immune modulation and development of myocarditis during *T. cruzi* infection (61), and some of the findings reported in their work correlate with our findings in AhRd mice. It has been reported that besides being refractory to most of the TCDD toxic effects, AhR KO mice exhibit multiple physiological abnormalities that are independent of xenobiotic exposure (62). The fact that AhR KO mice present an abnormal development of the immune system and the liver (63), should be an important constraint for evaluate the role of this receptor in modulating immune response and liver pathology in a model of infection in which the main target of pathological inflammation is the liver, and not the heart as in infected human patients or BALB/c mice. For that reason, we decided to evaluate the role of AhR activation by natural ligands (as Trp metabolites) generated during *T cruzi* infection by using B6-AhRd congenic mice. AhRd mice express an AhR with a 10-fold lower binding affinity for TCDD, and required a 10-fold higher dose of TCDD than AhRb-1 allele, present in B6 mice, to elicit similar effects (46). We have found that these mice suffer a more pronounced collapse of the Treg population than WT mice, which is consistent with weak/lack of AhR activation (3, 7). As expected, low Treg levels were associated to a stronger Th1 response which was able to control the parasite burden during the acute and the chronic phase of the infection. Significantly reduced parasitemia and cardiac pathology with increased levels of splenic IFN-γ-producing cells were also observed by Barroso el al. during the acute phase of AhR KO mice infected with Y strain of *T. cruzi*, although they not observed significant differences in Treg cells compared with WT mice (61). Surprisingly, in our experimental model AhRd mice were protected from the Th1-dependent liver pathology. Here we showed that these mice developed a Treg-independent mechanism of Th1 constriction between 10- and 17-days pi. After day 10 pi, serum levels of IL-10 were increased and spleen CD4+ cells secreting IL-10 were significantly up-regulated in AhRd vs. WT mice. Increased levels of CD4+ IL-10 producing T cells were also observed in AhR KO mice during the acute phase of *T. cruzi* infection (61). Likewise, IL-10 production by liver CD4+ T cells and increased levels of serum IL-10 were also found after *Plasmodium berghei* Anka infection in AhR KO mice (64). We speculate that the population of CD4+ IL-10-secreting T cells that is increased in *T. cruzi*-infected AhRd mice are different from Tr1 cells, because the *in vivo* generation of Tr1 cells is strongly dependent on AhR signals and these signals are very weak in AhRd mice (65). Moreover, in our model we have observed that Lag-3 [a marker of Tr1 cells (60)] is not up-regulated in the IL-10-producing CD4+ cells, and that many of these cells are also IFN-γ producers (data not shown). IL-10 production from effector T cells represents an essential negative feedback mechanism in the self-limitation of excessive inflammatory responses in many infections (66–69). Thus, the relative amounts of IL-10 and IFN-γ produced by Th1 cells may influence the balance between clearance, persistent chronic infection or immunopathology (70, 71). It has been demonstrated that IL-10 producing Th1 cells, also called self-regulatory Th1 cells, are activated early in a strong inflammatory environment by repeated TCR triggering (high antigen dose) and continued IL-12 action (72). Because fully differentiated Th1 cells fail to up-regulate AhR after activation and, therefore, cannot be directly modulated by AhR ligation, we hypothesize that weak/lack AhR activation in DCs induces differentiation signals that promote IL-10 production by CD4+ T cells. It has been demonstrated that AhR activation induces tolerogenic properties to DCs. In fact, Quintana and others have reported that AhR DC activation reduces the expression of the pro-inflammatory cytokines IL-1β, IL-6, and IL-12 (12, 13). In agreement, we have observed a higher IL-12 production by splenocytes from *T. cruzi*-infected AhRd vs. WT mice (data not shown), and this has also been reported in *T. cruzi*-infected and OVA-immunized AhR KO mice (61, 73).

Few studies have examined whether AhR signaling affects the acquisition of immunological memory, but several epidemiological studies have revealed that exposure to dioxins and PCBs conditions low response to routine childhood vaccinations (74–76). In accordance with this, our results demonstrated a negative effect of a strong AhR activation on the development of memory subsets, since the administration of single dose of TCDD before *T. cruzi* infection decreased the specific cellular response and prevented the accumulation of memory cells by a mechanism that involve the death of activated cells. Diminished memory response was also observed by TCDD administration before primary influenza virus infection, (47, 77). Even so, when TCDD is administered after the immunological memory is established, there are no detectable effects on the magnitude of the recall CD8+ T cell responses, suggesting that AhR activation modulates critical events for the activation of naïve T cells (47, 77), and as far as we know there are no reports about the effect of AhR activation with ligands other than TCDD on memory subsets development. Because antigen-experienced cells suffered the direct effect of TCDD toxicity, no conclusions can be drawn regarding the effects of AhR ligation on the development of memory subsets during the acute phase of *T. cruzi* infection. Therefore, we considered that weak AhR ligation with 3-HK plus ITE and AhRd mice are good models to study the development of CD8+ T cell memory subsets in this infection. Interestingly, 3HK plus ITE activation of AhR decreased the percentage of total and specific CD8+ splenic T cells that acquired EM and CM phenotype while the lack/very weak activation of AhR in AhRd mice showed opposite results. Certainly, at the chronic phase of the infection, AhRd mice had significantly higher frequency of splenic cells expressing IL-7 receptor (CD127), a marker for long-living memory T cells that would allow their vigorous proliferation driven by the homeostatic cytokine IL-7 (78). Together, these results suggest that AhR ligation restricts the differentiation of CD8+ memory T cells. Like other persistent infections, during *T. cruzi* infection the CD8+ T cells are maintained primary by the presence of antigen, and thus have phenotype of EM over CM as observed in transient infections (79). Interestingly, 3-HK plus ITE treated mice showed diminished number of EM cells compared with untreated mice, although the former have more parasite load able to drive preferably EM phenotype in CD8+ T cells, thus supporting the role of AhR activation on restricting memory development.

Different studies suggest that, similar to its indirect role on Th1 cells, AhR would likely regulate DCs rather than directly CD8+ T cells (47, 80). The very low expression of AhR in most CD8+ T cell populations might support the lack of direct regulation of CD8+ T cells by the AhR signaling. Thus, DCs may be the main cell population capable of sensing and secreting AhR ligands during *T. cruzi* infection, and the lack, weak or strong activation of DC AhR might modulate their activation status, inducing either proinflammatory or tolerogenic net effects on T cells and modulating memory subsets development (3, 4, 81, 82). Taken together, our results allow us to propose a model in which a threshold of AhR activation exist. Thus, signals above the threshold (corresponding to WT B6 mice) would induce tolerogenic properties in DCs (12, 13), restrict the development of memory CD8+ T cells and promote Treg cells (as observed here by treatment of B6 mice with 3-HK plus ITE or TCDD) able to prematurely control Th1-type response. On the contrary, signals below threshold would promote an early inflammatory Th1-type response able to restrict the parasite replication and its timely contraction by non-Tr1 IL-10 producing cells. Thus, AhR induce diverse regulatory pathways that finally impacts on parasite replication and infection outcome.

## Ethics Statement

All animal experiments were approved by and conducted in accordance with guidelines of the Institutional Animal Care and Use Committee (IACUC), Facultad de Ciencias Químicas, Universidad Nacional de Córdoba (Approval Number HCD 743/18).

## Author Contributions

LA, LC, and CM designed the experiments. LA, CI, XV, and EAR performed the experiments, data analysis and interpretation. LA, EAR, LC and CM wrote the manuscript. FJQ and HMS revised critically the manuscript.

### Conflict of Interest Statement

The authors declare that the research was conducted in the absence of any commercial or financial relationships that could be construed as a potential conflict of interest.

## References

[1] Quintana FJ Sherr DH. Aryl hydrocarbon receptor control of adaptive immunity. Pharmacol Rev. (2013) 65:1148–61. 10.1124/pr.113.007823 23908379 PMC3799235

[2] Kerkvliet NI. AHR-mediated immunomodulation: The role of altered gene transcription. Biochem Pharmacol. (2009) 77:746–60. 10.1016/j.bcp.2008.11.021 19100241 PMC2662368

[3] Mezrich JD Fechner JH Zhang X Johnson BP Burlingham WJ Bradfield CA. An Interaction between Kynurenine and the Aryl Hydrocarbon Receptor Can Generate Regulatory T Cells. J Immunol. (2010) 185:3190–8. 10.4049/jimmunol.0903670 20720200 PMC2952546

[4] Nguyen NT Kimura A Nakahama T Chinen I Masuda K Nohara K. Aryl hydrocarbon receptor negatively regulates dendritic cell immunogenicity via a kynurenine-dependent mechanism. Proc Natl Acad Sci USA. (2010) 107:19961–6. 10.1073/pnas.1014465107 21041655 PMC2993339

[5] Nguyen L Bradfield C. The search for endogenous activators of the aryl hydrocarbon receptor. Chem Res Toxicol. (2008) 21:102–16. 10.1021/tx7001965 18076143 PMC2572005

[6] Patel RD Murray IA Flaveny CA Kusnadi A Perdew GH. Ah receptor represses acute-phase response gene expression without binding to its cognate response element. Lab Investig. (2009) 89:695–707. 10.1038/labinvest.2009.24 19333233 PMC2743259

[7] Quintana F Basso A Iglesias A Korn T Farez M Bettelli E. Control of T(reg) and T(H)17 cell differentiation by the aryl hydrocarbon receptor. Nature. (2008) 453:65–71. 10.1038/nature06880 18362915

[8] Veldhoen M Hirota K Westendorf A Buer J Dumoutier L Renauld J. The aryl hydrocarbon receptor links TH17-cell-mediated autoimmunity to environmental toxins. Nature. (2008) 453:106–9. 10.1038/nature06881 18362914

[9] Kimura A Naka T Nohara K Fujii-Kuriyama Y Kishimoto T. Aryl hydrocarbon receptor regulates Stat1 activation and participates in the development of Th17 cells. Proc Natl Acad Sci USA. (2008) 105:9721–6. 10.1073/pnas.0804231105 18607004 PMC2474493

[10] Nguyen NT Nakahama T Kishimoto T. Aryl hydrocarbon receptor and experimental autoimmune arthritis. Semin Immunopathol. (2013) 35:637–44. 10.1007/s00281-013-0392-6 23982178

[11] Cibrian D Saiz ML de la Fuente H Sánchez-Díaz R Moreno-Gonzalo O Jorge I CD69 controls the uptake of L-tryptophan through LAT1-CD98 and AhR-dependent secretion of IL-22 in psoriasis. Nat Immunol. (2016) 17:985–96. 10.1038/ni.3504 27376471 PMC5146640

[12] Quintana FJ Murugaiyan G Farez MF Mitsdoerffer M Tukpah A-M Burns EJ From the Cover: An endogenous aryl hydrocarbon receptor ligand acts on dendritic cells and T cells to suppress experimental autoimmune encephalomyelitis. Proc Natl Acad Sci USA. (2010) 107:20768–73. 10.1073/pnas.1009201107 21068375 PMC2996442

[13] Gandhi R Kumar D Burns E Nadeau M Dake B Laroni A. Activation of the aryl hydrocarbon receptor induces human type 1 regulatory T cell-like and Foxp3(+) regulatory T cells. Nat Immunol. (2010) 11:846–53. 10.1038/ni.1915 20676092 PMC2929008

[14] Rothhammer V Mascanfroni ID Bunse L Takenaka MC Kenison JE Mayo L. Type I interferons and microbial metabolites of tryptophan modulate astrocyte activity and central nervous system inflammation via the aryl hydrocarbon receptor. Nat Med. (2016) 22:586–97. 10.1038/nm.4106 27158906 PMC4899206

[15] Salvatella R Irabedra P Sánchez D Castellanos LG Espinal M. South-south cooperation for Chagas disease. Lancet. (2013) 382:395–6. 10.1016/S0140-6736(13)61671-2 23911378

[16] Moretti E Basso B Cervetta L Brigada A Barbieri G. Patterns of cytokines and soluble cellular receptors in the sera of children with acute Chagas' disease. Clin Diagn Lab Immunol. (2002) 9:1324–7. 10.1128/CDLI.9.6.1324-1327.2002 12414768 PMC130093

[17] Lúcia CJA Luiz VR Bárbara I Fabiana A Fernando B Dirceu C Chronic Chagas' Disease Cardiomyopathy Patients Display an Increased IFN-γ Response to *Trypanosoma cruzi* Infection. J Autoimmun. (2001) 17:99–107. 10.1006/jaut.2001.0523 11488642

[18] Gomes JAS Bahia-Oliveira LMG Rocha MOC Martins-Filho OA Gazzinelli G Correa-Oliveira R. Evidence that Development of Severe Cardiomyopathy in Human Chagas' Disease Is Due to a Th1-Specific Immune Response. Infect Immun. (2003) 71:1185–93. 10.1128/IAI.71.3.1185-1193.2003 12595431 PMC148818

[19] Araujo FF Gomes J Rocha M Williams-Blangero S Pinheiro VM Morato M. Potential role of CD4+ CD25HIGH regulatory T cells in morbidity in Chagas disease. Front Biosci. (2006) 12:2797–806. 17485260 10.2741/2273

[20] da Silveira AB de AraÛjo FF Freitas MAR Gomes JAS Chaves AT de Oliveira EC. Characterization of the presence and distribution of Foxp3+ cells in chagasic patients with and without megacolon. Hum Immunol. (2009) 70:65–7. 10.1016/j.humimm.2008.10.015 19022313

[21] Araujo F Vitelli-Avelar D Teixeira-Carvalho A Antas P Gomes J Sathler-Avelar R. Regulatory T cells phenotype in different clinical forms of Chagas' disease. PLoS Negl Trop Dis. (2011) 5:992. 10.1371/journal.pntd.0000992 21655351 PMC3104959

[22] Guedes PMdM Gutierrez FRS Maia FL Milanezi CM Silva GK Pavanelli WR IL-17 produced during *Trypanosoma cruzi* infection plays a central role in regulating parasite-induced myocarditis. PLoS Negl Trop Dis. (2010) 4:e604 10.1371/journal.pntd.0000604 20169058 PMC2821906

[23] de Lourdes Higuchi M Gutierrez PS Aiello VD Palomino S Bocchi E Kalil J Immunohistochemical characterization of infiltrating cells in human chronic chagasic myocarditis: comparison with myocardial rejection process. Virchows Archiv A. (1993) 423:157–60. 10.1007/BF01614765 7901937

[24] Rocha Rodrigues DB dos Reis MA Romano A Pereira SA Teixeira Vde P Tostes S Jr. *In situ* expression of regulatory cytokines by heart inflammatory cells in Chagas' disease patients with heart failure. Clin Dev Immunol. (2012) 2012:361730. 10.1155/2012/361730 22811738 PMC3397162

[25] Cunha-Neto E Dzau VJ Allen PD Stamatiou D Benvenutti L Higuchi ML. Cardiac gene expression profiling provides evidence for cytokinopathy as a molecular mechanism in Chagas' disease cardiomyopathy. Am J Pathol. (2005) 167:305–13. 10.1016/S0002-9440(10)62976-8 16049318 PMC1603558

[26] Martins Reis M Higuchi MdL Benvenuti L Demarchi Aiello V Sampaio Gutierrez P Bellotti G An *in situ* quantitative immunohistochemical study of cytokines and IL-2R+ in chronic human chagasic myocarditis: correlation with the presence of myocardial *Trypanosoma cruzi* antigens. Clin Immunol Immunopathol. (1997) 83:165–72. 10.1006/clin.1997.4335 9143377

[27] Argüello RJ Vigliano C Cabeza-Meckert P Viotti R Garelli F Favaloro LE. Presence of antigen-experienced T cells with low grade of differentiation and proliferative potential in chronic Chagas disease myocarditis. PLoS Negl Trop Dis. (2014) 8:e2989. 10.1371/journal.pntd.0002989 25144227 PMC4140664

[28] Junqueira C Caetano B Bartholomeu DC Melo MB Ropert C Rodrigues MM. The endless race between Trypanosoma cruzi and host immunity: lessons for and beyond Chagas disease. Exp Rev Mol Med. (2010) 12:e29. 10.1017/S1462399410001560 20840799

[29] Roggero E Perez A Tamae-Kakazu M Piazzon I Nepomnaschy I Wietzerbin J Differential susceptibility to acute Trypanosoma cruzi infection in BALB/c and C57BL/6 mice is not associated with a distinct parasite load but cytokine abnormalities. Clin Exp Immunol. (2002) 128:421–8. 10.1046/j.1365-2249.2002.01874.x 12067296 PMC1906265

[30] González FB Villar SR Fernández Bussy R Martin GH Pérol L Manarin R. Immunoendocrine dysbalance during uncontrolled *T. cruzi* infection is associated with the acquisition of a Th-1-like phenotype by Foxp3(+) T cells. Brain Behav Immun. (2015) 45:219–32. 10.1016/j.bbi.2014.11.016 25483139 PMC7126853

[31] Araujo Furlan CL Tosello Boari J Rodriguez C Canale FP Fiocca Vernengo F Boccardo S. Limited Foxp3(+) Regulatory T Cells Response During Acute *Trypanosoma cruzi* Infection Is Required to Allow the Emergence of Robust Parasite-Specific CD8(+) T Cell Immunity. Front Immunol. (2018) 9:2555. 10.3389/fimmu.2018.02555 30455700 PMC6230662

[32] Knubel CP Martinez FF Fretes RE Lujan CD Theumer MG Cervi L. Indoleamine 2,3-dioxigenase (IDO) is critical for host resistance against Trypanosoma cruzi. FASEB J. (2010) 24:2689–701. 10.1096/fj.09-150920 20233946

[33] Knubel CP Insfran C Martinez FF Diaz Lujan C Fretes RE Theumer MG. 3-Hydroxykynurenine, a Tryptophan Metabolite Generated during the Infection, Is Active Against *Trypanosoma cruzi*. ACS Med Chem Lett. (2017) 8:757–61. 10.1021/acsmedchemlett.7b00169 28740612 PMC5512135

[34] Knubel CP Martinez FF Acosta Rodriguez EV Altamirano A Rivarola HW Diaz Luja¡n C. 3-Hydroxy kynurenine treatment controls *T. Cruzi* replication and the inflammatory pathology preventing the clinical symptoms of chronic chagas disease. PLoS ONE. (2011) 6:e26550. 10.1371/journal.pone.0026550 22028903 PMC3197528

[35] Vogel CF Goth SR Dong B Pessah IN Matsumura F. Aryl hydrocarbon receptor signaling mediates expression of indoleamine 2,3-dioxygenase. Biochem Biophys Res Commun. (2008) 375:331–5. 10.1016/j.bbrc.2008.07.156 18694728 PMC2583959

[36] Hassanain HH Chon SY Gupta SL. Differential regulation of human indoleamine 2,3-dioxygenase gene expression by interferons-gamma and -alpha. Analysis of the regulatory region of the gene and identification of an interferon-gamma-inducible DNA-binding factor. J Biol Chem. (1993) 268:5077–84. 8444884

[37] Bessede A Gargaro M Pallotta MT Matino D Servillo G Brunacci C. Aryl hydrocarbon receptor control of a disease tolerance defence pathway. Nature. (2014) 511:184. 10.1038/nature13323 24930766 PMC4098076

[38] Piron M Fisa R Casamitjana N Lopez-Chejade P Puig L Verges M. Development of a real-time PCR assay for *Trypanosoma cruzi* detection in blood samples. Acta Trop. (2007) 103:195–200. 10.1016/j.actatropica.2007.05.019 17662227

[39] Funatake CJ Marshall NB Steppan LB Mourich DV Kerkvliet NI. Cutting Edge: Activation of the Aryl Hydrocarbon Receptor by 2,3,7,8-Tetrachlorodibenzo-p-dioxin Generates a Population of CD4+CD25+ Cells with Characteristics of Regulatory T Cells. J Immunol. (2005) 175:4184–8. 10.4049/jimmunol.175.7.4184 16177056

[40] Veiga-Parga T Suryawanshi A Rouse BT. Controlling viral immuno-inflammatory lesions by modulating Aryl hydrocarbon receptor signaling. PLoS Pathog. (2011) 7:e1002427. 10.1371/journal.ppat.1002427 22174686 PMC3234248

[41] Benson JM Shepherd DM. Aryl hydrocarbon receptor activation by TCDD reduces inflammation associated with Crohn's disease. Toxicol Sci. (2011) 120:68–78. 10.1093/toxsci/kfq360 21131560 PMC3044199

[42] Martin DL Weatherly DB Laucella SA Cabinian MA Crim MT Sullivan S. CD8+ T-Cell responses to Trypanosoma cruzi are highly focused on strain-variant trans-sialidase epitopes. PLoS Pathog. (2006) 2:e77. 10.1371/journal.ppat.0020077 16879036 PMC1526708

[43] Yamaguchi T Hirota K Nagahama K Ohkawa K Takahashi T Nomura T. Control of immune responses by antigen-specific regulatory T cells expressing the folate receptor. Immunity. (2007) 27:145–59. 10.1016/j.immuni.2007.04.017 17613255

[44] Nugent LF Shi G Vistica BP Ogbeifun O Hinshaw SJH Gery I. ITE, a novel endogenous nontoxic Aryl hydrocarbon receptor ligand, efficiently suppresses EAU and T-cell–mediated immunity. Invest Ophthalmol Visual Sci. (2013) 54:7463–9. 10.1167/iovs.12-11479 24150760 PMC3828045

[45] Yeste A Takenaka MC Mascanfroni ID Nadeau M Kenison JE Patel B. Tolerogenic nanoparticles inhibit T cell–mediated autoimmunity through SOCS2. Sci Signal. (2016) 9:ra61-ra. 10.1126/scisignal.aad0612 27330188

[46] Poland A Palen D Glover E. Analysis of the four alleles of the murine aryl hydrocarbon receptor. Mol Pharmacol. (1994) 46:915–21. 7969080

[47] Lawrence BP Roberts AD Neumiller JJ Cundiff JA Woodland DL Aryl hydrocarbon receptor activation impairs the priming but not the recall of influenza virus-specific CD8+ T cells in the lung. J Immunol. (2006) 177:5819–28. 10.4049/jimmunol.177.9.5819 17056506

[48] Lawrence BP Vorderstrasse B. New insights into the aryl hydrocarbon receptor as a modulator of host responses to infection. Semin Immunopathol. (2013) 35:615–26. 10.1007/s00281-013-0395-3 23963494 PMC3808126

[49] Shinde R McGaha TL. The Aryl hydrocarbon receptor: connecting immunity to the microenvironment. Trends Immunol. (2018) 39:1005–20. 10.1016/j.it.2018.10.010 30409559 PMC7182078

[50] Singh NP Nagarkatti M Nagarkatti P Primary peripheral T cells become susceptible to 2, 3, 7, 8-Tetrachlorodibenzo-p-Dioxin (TCDD)-mediated apoptosis *in vitro* upon activation and in the presence of dendritic cells. Mol Pharmacol. (2008) 73:1722–35. 10.1124/mol.107.043406 18334599 PMC2828294

[51] Winans B Nagari A Chae M Post CM Ko C-I Puga A. Linking the Aryl hydrocarbon receptor with altered DNA methylation patterns and developmentally induced aberrant antiviral CD8 T cell responses. J Immunol. (2015) 194:4446. 10.4049/jimmunol.1402044 25810390 PMC4402273

[52] Winzler C Fantinato M Giordan M Calore E Basso G Messina C. CD4+ T regulatory cells are more resistant to DNA damage compared to CD4+ T effector cells as revealed by flow cytometric analysis. Cytometry Part A. (2011) 79:903–11. 10.1002/cyto.a.21132 22015731

[53] Fritzsching B Oberle N Eberhardt N Quick S Haas J Wildemann B Cutting edge: in contrast to effector T cells, CD4+ CD25+ Foxp3+ regulatory T cells are highly susceptible to CD95 ligand-but not to TCR-mediated cell death. J Immunol. (2005) 175:32–6. 10.4049/jimmunol.175.1.32 15972628

[54] Banz A Pontoux C Papiernik M. Modulation of fas-dependent apoptosis: a dynamic process controlling both the persistence and death of CD4 regulatory T cells and effector T cells. J Immunol. (2002) 169:750–7. 10.4049/jimmunol.169.2.750 12097377

[55] Che JW Kraft ARM Selin LK Welsh RM. Regulatory T cells resist virus infection-induced apoptosis. J Virol. (2015) 89:2112–20. 10.1128/JVI.02245-14 25473049 PMC4338871

[56] Silva JS Morrissey PJ Grabstein KH Mohler KM Anderson D Reed SG. Interleukin 10 and interferon gamma regulation of experimental Trypanosoma cruzi infection. J Exp Med. (1992) 175:169–74. 10.1084/jem.175.1.169 1730915 PMC2119081

[57] DeKrey GK Teagarden RE Lenberg JL Titus RG. 2,3,7,8-Tetrachlorodibenzo-p-dioxin slows the progression of experimental cutaneous leishmaniasis in susceptible BALB/c and SCID mice. PLoS ONE. (2013) 8:e76259. 10.1371/journal.pone.0076259 24098456 PMC3788076

[58] Bowers OJ Sommersted KB Sowell RT Boling GE Hanneman WH Titus RG. 2, 3, 7, 8-tetrachlorodibenzo-p-dioxin (TCDD) reduces Leishmania major burdens in C57BL/6 mice. Am J Trop Med Hygiene. (2006) 75:749–52. 10.4269/ajtmh.2006.75.749 17038706

[59] Apetoh L Quintana F Pot C Joller N Xiao S Kumar D. The aryl hydrocarbon receptor interacts with c-Maf to promote the differentiation of type 1 regulatory T cells induced by IL-27. Nat Immunol. (2010) 11:854–61. 10.1038/ni.1912 20676095 PMC2940320

[60] Gagliani N Magnani CF Huber S Gianolini ME Pala M Licona-Limon P. Coexpression of CD49b and LAG-3 identifies human and mouse T regulatory type 1 cells. Nat Med. (2013) 19:739–46. 10.1038/nm.3179 23624599

[61] Barroso A Gualdrón-López M Esper L Brant F Araújo RR Carneiro MB. The aryl hydrocarbon receptor modulates production of cytokines and reactive oxygen species and development of myocarditis during Trypanosoma cruzi infection. Infect Immun. (2016) 84:3071–82. 10.1128/IAI.00575-16 27481250 PMC5038084

[62] McMillan BJ Bradfield CA. The Aryl Hydrocarbon receptor sans xenobiotics: endogenous function in genetic model systems. Mol Pharmacol. (2007) 72:487–98. 10.1124/mol.107.037259 17535977

[63] Fernandez-Salguero P Pineau T Hilbert D McPhail T Lee S Kimura S. Immune system impairment and hepatic fibrosis in mice lacking the dioxin-binding Ah receptor. Science. (1995) 268. :722–6. 10.1126/science.7732381 7732381

[64] Brant F Miranda AS Esper L Rodrigues DH Kangussu LM Bonaventura D. Role of the aryl hydrocarbon receptor in the immune response profile and development of pathology during *Plasmodium berghei* Anka infection. Infect Immun. (2014) 82:3127–40. 10.1128/IAI.01733-14 24818665 PMC4136209

[65] Wu HY Quintana FJ da Cunha AP Dake BT Koeglsperger T Starossom SC. *In vivo* induction of Tr1 cells via mucosal dendritic cells and AHR signaling. PLoS ONE. (2010) 6:e23618. 10.1371/journal.pone.0023618 21886804 PMC3160310

[66] Sun J Madan R Karp CL Braciale TJ. Effector T cells control lung inflammation during acute influenza virus infection by producing IL-10. Nat Med. (2009) 15:277–84. 10.1038/nm.1929 19234462 PMC2693210

[67] O'Garra A Vieira P TH1 cells control themselves by producing interleukin-10. Nat Rev Immunol. (2007) 7:425–8. 10.1038/nri2097 17525751

[68] Anderson CF Oukka M Kuchroo VJ Sacks D. CD4+ CD25– Foxp3– Th1 cells are the source of IL-10–mediated immune suppression in chronic cutaneous leishmaniasis. J Exp Med. (2007) 204:285–97. 10.1084/jem.20061886 17283207 PMC2118728

[69] Jankovic D Kullberg MC Feng CG Goldszmid RS Collazo CM Wilson M. Conventional T-bet+ Foxp3– Th1 cells are the major source of host-protective regulatory IL-10 during intracellular protozoan infection. J Exp Med. (2007) 204:273–83. 10.1084/jem.20062175 17283209 PMC2118735

[70] Moore KW de Waal Malefyt R Coffman RL O'Garra A. Interleukin-10 and the interleukin-10 receptor. Annu Rev Immunol. (2001) 19:683–765. 10.1146/annurev.immunol.19.1.683 11244051

[71] Trinchieri G. Interleukin-10 production by effector T cells: Th1 cells show self control. J Exp Med. (2007) 204:239–43. 10.1084/jem.20070104 17296790 PMC2118719

[72] Saraiva M Christensen JR Veldhoen M Murphy TL Murphy KM O'Garra A. Interleukin-10 production by Th1 cells requires interleukin-12-induced STAT4 transcription factor and ERK MAP kinase activation by high antigen dose. Immunity. (2009) 31:209–19. 10.1016/j.immuni.2009.05.012 19646904 PMC2791889

[73] Rodríguez-Sosa M Elizondo G López-Durán RM Rivera I Gonzalez FJ Vega L. Over-production of IFN-γ and IL-12 in AhR-null mice. FEBS Lett. (2005) 579:6403–10. 10.1016/j.febslet.2005.10.023 16289099

[74] Heilmann C Grandjean P Weihe P Nielsen F Budtz-Jørgensen E. Reduced antibody responses to vaccinations in children exposed to polychlorinated biphenyls. PLoS Med. (2006) 3:e311. 10.1371/journal.pmed.0030311 16942395 PMC1551916

[75] Stølevik SB Nygaard UC Namork E Haugen M Meltzer HM Alexander J. Prenatal exposure to polychlorinated biphenyls and dioxins from the maternal diet may be associated with immunosuppressive effects that persist into early childhood. Food Chem Toxicol. (2013) 51:165–72. 10.1016/j.fct.2012.09.027 23036451

[76] Hochstenbach K van Leeuwen DM Gmuender H Gottschalk RW Stølevik SB Nygaard UC. Toxicogenomic profiles in relation to maternal immunotoxic exposure and immune functionality in newborns. Toxicol Sci. (2012) 129:315–24. 10.1093/toxsci/kfs214 22738990 PMC3529642

[77] Lawrence BP Vorderstrasse BA. Activation of the Aryl hydrocarbon receptor diminishes the memory response to homotypic influenza virus infection but does not impair host resistance. Toxicol Sci. (2004) 79:304–14. 10.1093/toxsci/kfh094 14976337

[78] Prlic M Lefrancois L Jameson SC. Multiple choices: regulation of memory CD8 T cell generation and homeostasis by interleukin (IL)-7 and IL-15. J Exp Med. (2002) 195:F49–52. 10.1084/jem.20020767 12070294 PMC2193558

[79] Padilla AM Bustamante JM Tarleton RL. CD8+ T cells in Trypanosoma cruzi infection. Curr Opin Immunol. (2009) 21:385–90. 10.1016/j.coi.2009.07.006 19646853 PMC2735075

[80] Jin G-B Moore AJ Head JL Neumiller JJ Lawrence BP. Aryl hydrocarbon receptor activation reduces dendritic cell function during influenza virus infection. Toxicol Sci. (2010) 116:514–22. 10.1093/toxsci/kfq153 20498003 PMC2905408

[81] Vogel CF Wu D Goth SR Baek J Lollies A Domhardt R. Aryl hydrocarbon receptor signaling regulates NF-κB RelB activation during dendritic-cell differentiation. Immunol Cell Biol. (2013) 91:568–75. 10.1038/icb.2013.43 23999131 PMC3806313

[82] Kuo CHH Hsieh CCC Kuo HFF Huang MYY Yang SNN Chen LCC. Phthalates suppress type I interferon in human plasmacytoid dendritic cells via epigenetic regulation. Allergy. (2013) 68:870–9. 10.1111/all.12162 23738920

