# Supplementary material for: Role of Aryl Hydrocarbon Receptor (AhR) in the Regulation of Immunity and Immunopathology During Trypanosoma cruzi Infection
Source: Front Immunol. 2019 Mar 29;10:631. doi: 10.3389/fimmu.2019.00631 (PMC6450169; doi:10.3389/fimmu.2019.00631)
Supplement: Supplementary file 1 [file Data_Sheet_1.docx]

Supplementary Material

Role of Aryl Hydrocarbon Receptor (AhR) in the regulation of Immunity and Immunopathology During *Trypanosoma cruzi* Infection.

**Laura Fernanda Ambrosio^1,2^, Constanza Insfran^1,2^, Ximena Volpini^1,2^, Eva Acosta Rodriguez^1,2^, Horacio Marcelo Serra^1,2^, Francisco J. Quintana^3,4^, Laura Cervi^1,2^ and Claudia Cristina Motrán^1,2*^.**

*** Correspondence:** Corresponding Author: [cmotran@fcq.unc.edu.ar](mailto:cmotran@fcq.unc.edu.ar)

**A**

**B**

**Figure S1. Combined 3-HK + ITE treatment did not impact on parasitemia or survival.** C57BL/6J mice were infected with 50,000 Tps of *T. cruzi* and then i.p injected with 3-HK + ITE or vehicle as control as indicated in Figure 3A. A) Parasitemia (Tps/ml blood). Data are shown as mean ± SD, n= 4 mice per group B) Survival rate of *T. cruzi* infected 3-HK + ITE and control mice, n= 6 mice per group. *P* values were calculated with the Gehan-Breslow-Wilcoxon test.


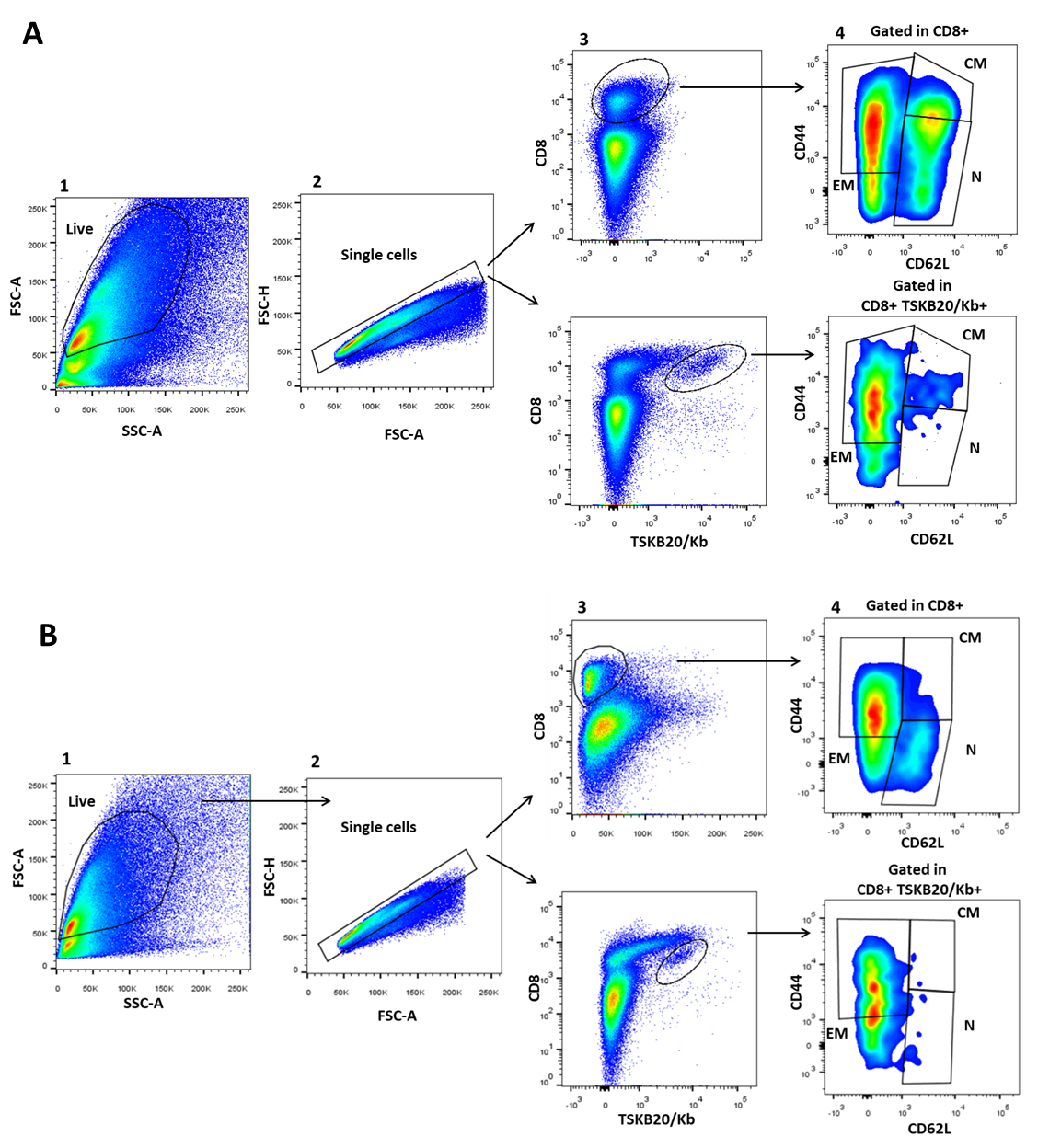


**Figure S2. Gating strategy for CD8+ and CD8+ TSKB20/Kb+ memory populations in TCDD- and 3-HK + ITE-treated mice spleen cells** Representative dot plots of the gating strategy used to analyze memory populations in TCDD- (A) 3-HK + ITE-treated (B) mice and their respective control groups. Cells were first gated in a region considered “live” according to size and complexity (FSC-H vs. FSC-A) (1). Then they were gated for singlets (FSC-H vs. FSC-A) (2) and subsequently CD8+ (upper dotplot) and CD8+ TSKB20+ (lower dotplot) (3). Finally CD44 and CD62L markers were used in the previously gated populations to identify central memory (CM, CD44+ CD62L+), effector/effector memory (EM, CD44+ CD62L-) and naïve (N, CD44- CD62L+) (4).


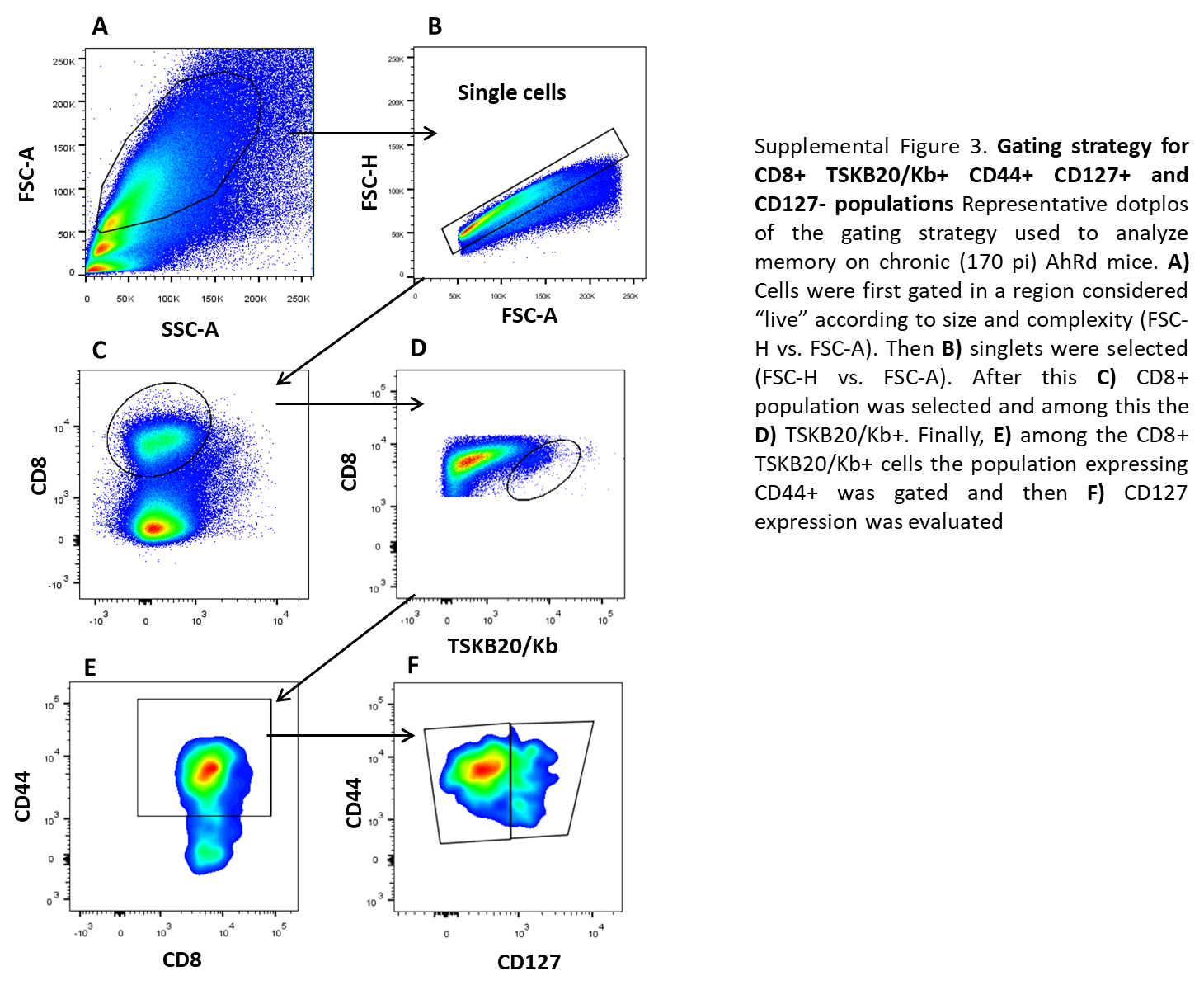


**Figure S3. Gating strategy for CD8+ TSKB20/Kb+ CD44+ CD127+ and CD127- populations.** Representative dotplos of the gating strategy used to analyze memory on chronic (170 pi) AhRd mice. A) Cells were first gated in a region considered “live” according to size and complexity (FSC-H vs. FSC-A). Then B) singlets were selected (FSC-H vs. FSC-A). After this C) CD8+ population was selected and among this the D) TSKB20/Kb+. Finally, E) among the CD8+ TSKB20/Kb+ cells the population expressing CD44+ was gated and then F) CD127 expression was evaluated
